# EchinoDB: an update to the web-based application for genomic and transcriptomic data on echinoderms

**DOI:** 10.1186/s12863-022-01090-6

**Published:** 2022-10-23

**Authors:** Varnika Mittal, Robert W. Reid, Denis Jacob Machado, Vladimir Mashanov, Daniel A. Janies

**Affiliations:** 1 Department of Bioinformatics and Genomics, College of Computing and Informatics, University of North Carolina at Charlotte, 9331 Robert D. Snyder Rd, Charlotte, NC 28223 USA; 2 Wake Forest Institute for Regenerative Medicine, 91 Technology Way NE, Winston-Salem, NC 27101 USA

**Keywords:** Database, Echinoderms, Echinoids, Gene family, Genome, Ophiuroids, Orthocluster, Ortholog, Paralog, Transcriptome, Notch, Wnt, Spicule matrix proteins, Mutable collagenous tissue, Tensilin

## Abstract

**Background:**

Here we release a new version of EchinoDB, EchinoDB v2.0 (https://echinodb.uncc.edu). EchinoDB is a database of genomic and transcriptomic data on echinoderms. The initial database consisted of groups of 749,397 orthologous and paralogous transcripts arranged in orthoclusters by sequence similarity.

**Results:**

The updated version of EchinoDB includes two new major datasets: the RNA-Seq data of the brittle star *Ophioderma brevispinum* and the high-quality genomic assembly data of the green sea urchin *Lytechinus variegatus*. In addition, we enabled keyword searches for annotated data and installed an updated version of Sequenceserver to allow Basic Local Alignment Search Tool (BLAST) searches. The data are downloadable in FASTA format. The first version of EchinoDB appeared in 2016 and was implemented in GO on a local server. The new version has been updated using R Shiny to include new features and improvements in the application. Furthermore, EchinoDB now runs entirely in the cloud for increased reliability and scaling.

**Conclusion:**

EchinoDB serves a user base drawn from the fields of phylogenetics, developmental biology, genomics, physiology, neurobiology, and regeneration. As use cases, we illustrate the function of EchinoDB in retrieving components of signaling pathways involved in the tissue regeneration process of different echinoderms, including the emerging model species *Ophioderma brevispinum*. Moreover, we use EchinoDB to shed light on the conservation of the molecular components involved in two echinoderm-specific phenomena: spicule matrix proteins involved in the formation of stereom endoskeleton and the tensilin protein that contributes to the capacity of the connective tissues to quickly change its mechanical properties. The genes involved in the former had been previously studied in echinoids, while gene sequences involved in the latter had been previously described in holothuroids. Specifically, we ask (a) if the biomineralization-related proteins previously reported only in sea urchins are also present in other, non-echinoid, echinoderms and (b) if tensilin, the protein responsible for the control of stiffness of the mutable collagenous tissue, previously described in sea cucumbers, is conserved across the phylum.

**Supplementary Information:**

The online version contains supplementary material available at 10.1186/s12863-022-01090-6.

## Background

The phylum Echinodermata is composed of marine invertebrate animals commonly known as echinoderms. It contains five extant classes: Asteroidea, Ophiuroidea, Holothuroidea, Echinoidea, and Crinoidea [1]. Echinoderms share a number of unique characteristics such as: pentaradial body symmetry (or modifications thereof) in adults, a skeleton composed of numerous ossicles formed of stereom (a calcium carbonate material), a water-vascular system, and a mutable collagenous tissue [2–5]. However, the most astonishing feature of echinoderms is their capacity to regenerate complex internal organs following injury or autotomy [6–13]. For instance, sea cucumbers (Echinodermata: Holothuroidea) have the ability to fully regenerate their digestive tube following visceral autotomy (evisceration) [14] and their radial nerve cord following transection [15]. Similarly, brittle stars of the class Ophiuroidea display remarkable regenerative capabilities in arm regeneration post injury or autotomy [16]. Regeneration in these animals involves substantial cell division, but it never goes awry to result in tumor formation [17]. Therefore, EchinoDB provides an opportunity to investigate genes involved in the evolution of echinoderm-specific traits (e.g., stereom skeleton and mutable collagenous tissue) and to deeply study fundamental genomic regulatory mechanisms underlying regeneration.

Researchers motivated by the biomedical potential of echinoderms have assembled a number of resources to study these animals. However current resources are limited to only a small fraction of species that do not represent the diversity within the phylum. Hence, to fill this gap, we have created EchinoDB, a database resource, in which genomic and transcriptomic data on 42 unique echinoderm species, spanning the deepest divergences within the five extant classes, is wrapped in an easy-to-use web-based application [18]. These species and associated raw sequence resources are listed in Table 1 and Additional file 1: Table S1. Our database thus allows for deep phylogenetic sampling within the echinoderm clade to facilitate data retrieval (annotated sequences) for various downstream projects, including regeneration, phylogeny, and gene family studies.

**Table 1:** Raw reads from the various echinoderm species that are available in NCBI’s SRA and Zenodo (doi: 10.5281/zenodo.6985492). Each line corresponds to transcriptome or gene expression data. Orthoclusters: number of orthoclusters. Sequences: number of amino acids or coding sequences. Length: sum of base pairs in all sequences. See complete table in Additional file 1: Table S1

Class: Order: Family	Species	Accession	SRR	Orthoclusters	Sequences	Length
Crinoidea: Comatulida: Zenometridae	*Psathryometra fragilis*	PRJNA299480	SRR2846085	6651	9015	3.16E+ 07
Asteroidea: Velatida: Xyloplacidae	*Xyloplax* sp. *Janetae* (BJ2)	PRJNA299326	SRR2846120	17,993	24,452	5.65E+ 07
Asteroidea: Spinulosida: Echinasteridae	*Echinaster spinulosus*	PRJNA300370	SRR2844624	13,844	18,608	6.41E+ 07
Ophiuroidea: Ophiocomidea: Ophiocomidae	*Ophiocoma wendtii*	PRJNA299897	SRR2845427	3662	9783	8.82E+ 07
Ophiuroidea: Gnathophiuridea: Ophiotrichidae	*Ophiothrix spiculata*	PRJNA299898	SRR2845448	8118	18,816	7.34E+ 07
Asteroidea: Velatida: Pterasteridae	*Pteraster tesselatus*	PRJNA299398	SRR2846094	46,531	51,762	1.71E+ 08
Holothuroidea: Apodida: Synaptidae	*Synapta maculata*	PRJNA299890	SRR2846103	5309	11,154	8.44E+ 07
Echinoidea: Echinoida: Strongylocentrotidae	*Strongylocentrotus purpuratus*	PRJNA299888	SRR2846101	6885	11,368	4.15E+ 07
Asteroidea: Forcipulatida: Asteriidae	*Pisaster ochraceus*	PRJNA299406	SRR2846074	37,807	43,479	1.68E+ 08
Holothuroidea: Dendrochirotida: Psolidae	*Psolus* sp. (BJ11)	PRJNA299550	NA	24,634	35,310	1.91E+ 08
Holothuroidea: Aspidochirotida: Stichopodidae	*Stichopus chloronotus*	PRJNA299896	SRR2846098	17,953	24,854	1.09E+ 08
Crinoidea: Comatulida: Colobometridae	*Oligometra serripinna*	PRJNA299464	SRR2845419	55,472	70,278	2.11E+ 08
Crinoidea: Comatulida: Bourgueticrinidae	*Democrinus brevis*	PRJNA299465	SRR2844622	6285	8287	4.72E+ 07
Asteroidea: Velatida: Korethrasteridae	*Peribolaster folliculatus* (BJ19)	PRJNA299409	SRR2845673	16,927	20,462	8.32E+ 07
Asteroidea: Paxillosida: Astropectinidae	*Psilaster charcoti*	PRJNA299410	SRR2846092	24,055	28,413	9.41E+ 07
Asteroidea: Forcipulatida: Labidiasteridae	*Labidiaster annulatus*	PRJNA299411	SRR2845003	35,615	40,071	1.43E+ 08
Asteroidea: Velatida: Korethrasteridae	*Remaster gourdoni*	PRJNA299412	SRR2846097	18,288	22,056	8.21E+ 07
Crinoidea: Hyocrinida: Hyocrinidae	*Gephyrocrinus messingi*	PRJNA300546	SRR2859800	8950	12,234	4.42E+ 07
Asteroidea: Paxillosida: Luidiidae	*Luidia clathrata*	PRJNA299414	SRR2845324	36,915	77,487	9.42E+ 07
Asteroidea: Spinulosida: Echinasteridae	*Henricia leviuscula* A	PRJNA299415	SRR2844627	47,492	76,684	9.58E+ 07
Asteroidea: Paxillosida: Astropectinidae	*Astropecten duplicatus*	PRJNA299417	SRR2843238	42,051	73,744	9.13E+ 07
Asteroidea: Valvatida: Poraniidae	*Glabraster antarctica* (BJ28)	PRJNA299418	SRR2844625	28,408	54,328	7.71E+ 07
Asteroidea: Valvatida: Asteropseidae	*Asteropsis carinifera*	PRJNA299419	SRR2843236	25,973	49,607	6.51E+ 07
Asteroidea: Valvatida: Solasteridae	*Peribolaster folliculatus* (BJ30*)*	PRJNA299409	SRR2845673	22,319	36,551	5.25E+ 07
Asteroidea: Notomyotida: Benthopectinidae	*Cheiraster hirsutus*	PRJNA299420	SRR2844620	325	1271	6.85E+ 06
Asteroidea: Brisingida: Brisingidae	*Odinella nutrix*	PRJNA299463	SRR2845408	312	1004	6.83E+ 06
Crinoidea: Comatulida: Ptilometridae	*Ptilometra australis*	PRJNA299466	SRR2846095	33,084	49,470	7.31E+ 07
Crinoidea: Comatulida: Comasteridae	*Cenolia new species*	PRJNA299468	SRR2847917	11,658	18,875	3.51E+ 07
Crinoidea: Comatulida: Antedonidae	*Isometra vivipara*	PRJNA299471	SRR2844835	27,204	43,689	7.02E+ 07
Crinoidea: Comatulida: Antedonidae	*Phrixometra nutrix*	PRJNA299469	SRR2846073	4923	12,283	2.83E+ 07
Crinoidea: Comatulida: Antedonidae	*Promachocrinus kerguelensis*	PRJNA299478	SRR2846076	8011	12,283	2.83E+ 07
Echinoidea: Arbacioida: Arbaciidae	*Arbacia punctulata*	PRJNA299547	SRR2843235	13,324	33,220	4.86E+ 07
Echinoidea: Cidaroida: Cidaridae	*Eucidaris tribuloides*	PRJNA299548	SRR2844624	6939	16,512	2.97E+ 07
Echinoidea: Clypeasteroida: Dendrasteridae	*Dendraster excentricus*	PRJNA299549	SRR2844623	4619	12,561	6.57E+ 07
Holothuroidea: Dendrochirotacea: Psolidae	*Psolus* sp. (BJ41)	PRJNA299550	NA	16,398	33,062	7.32E+ 07
Holothuroidea: Aspidochirotida: Synallactidae	*Peniagone* sp. (BJ42)	PRJNA299551	NA	12,286	22,457	5.25E+ 07
Holothuroidea: Dendrochirotacea: Cucumariidae	*Abyssocucumis* sp. (BJ43)	PRJNA299552	SRR2830762	12,309	26,171	5.47E+ 07
Holothuroidea: Aspidochirotida: Synallactidae	*Pseudostichopus* sp. (BJ44)	PRJNA299883	NA	2464	5567	1.36E+ 07
Holothuroidea: Molpadida: Molpadidae	*Molpadia intermedia*	PRJNA299884	SRR2845419	3793	6516	1.53E+ 07
Holothuroidea: Elasipodida: Laetmogonidae	*Pannychia moseleyi*	PRJNA299885	NA	10,124	20,051	3.96E+ 07
Ophiuroidea: Euryalida: Gorgonocephalidae	*Astrophyton muricatum*	PRJNA299886	SRR2843239	11,730	26,889	7.31E+ 07
Ophiuroidea: Ophiurida: Ophiodermatidae	*Ophioderma brevispinum*	PRJNA299887	SRR2845428	11,757	28,450	6.52E+ 07

EchinoDB v2.0 is an open-source web-based application (https://echinodb.uncc.edu), designed to provide genomic, transcriptomic and amino acid sequence data on echinoderms. The code for EchinoDB v2.0 is provided in Additional file 5: File S4.

The objective of EchinoDB is to serve research communities by providing diverse and rich data for a wide diversity of echinoderm species. The previous version of EchinoDB was released in 2016 and consisted of amino acid sequence orthoclusters (orthologous genes) from 42 echinoderm transcriptomes [19]. The new version has now been extended to incorporate new datasets that have been generated since the original release. These new datasets include RNA-Seq data for the brittle star *O. brevispinum* (Say, 1825) (Echinodermata: Ophiuroidea: Ophiacanthida: Ophiodermatidae) [16], genome assembly data of the green sea urchin *Lytechinus variegatus* (Lamarck, 1816) (Echinodermata: Echinoidea: Camarodonta: Toxopneustidae) [20], and phylogenomic data for *Xyloplax* sp. (Echinodermata: Asteroidea) [21]. The RNA-Seq data of the brittle star and the genome assembly data of the green sea urchin form the basis of two newly developed tools, OphiuroidDB [22] and EchinoidDB [23], respectively, integrated within the EchinoDB application.

An effective bioinformatics resource must keep up with new data, advances in software, server architecture, and programming languages. The need to improve reliability and scale well with the increasing amount of data and the number of users warranted an update to EchinoDB. The updated EchinoDB has been rewritten in R Shiny [24] and runs entirely in the cloud environment (AWS) [25]. R Shiny is highly extensible, easy to code and maintain, as compared to the previous implementation built using GO programming language in 2016. R Shiny supports faster development of user interfaces by providing a framework that requires no or little knowledge of scripting languages like HTML, CSS or JavaScript. We have taken advantage of this feature to extend the application’s capabilities to make new data (obtained from collaborations) easily available to the research community, for example, implementing the BLAST [26] search interface for the *Lytechinus* [20] and *Ophioderma* [16] sequences via Sequenceserver [27].

To demonstrate the practical utility of the new version of EchinoDB [18] and its associated resources - OphiuroidDB [22] and EchinoidDB [23] – we illustrate how EchinoDB is used in retrieving key components of the Notch and Wnt signaling pathways, that are crucial for tissue regeneration in echinoderms [16, 28–32]. In addition, we describe the use of SequenceServer (BLAST tool) [27, 33, 26] integrated within EchinoDB to find the putative homologs of the skeleton matrix proteins [4, 34–37] and tensilin (a protein that controls tensile strength of mutable collagenous tissues) [5, 38–40, 41, 42], previously reported in sea urchins (Echinodermata:Echinoidea) and sea cucumbers (Echinodermata: Holothuroidea).

## Construction and content

EchinoDB is re-factored in R Shiny and currently supports annotated transcriptomic data for 42 echinoderm species (see Table 1 or Additional file 1: Table S1), functional transcriptomic data from a Notch pathway inhibition study in *O. brevispinum* [16], and protein sequences from a chromosome-level genome assembly of *L. variegatus* [20]. R Shiny is highly extensible, that is, code developed with R Shiny can be readily integrated with CSS themes, HTML widgets, and scripting languages (e.g. JavaScript). In addition, R Shiny is widely adopted and the code can be modified and tuned at later stages in the development cycle by many developers. EchinoDB v2.0 is hosted using the Nginx web server [43] in Amazon Web Services (AWS) [25]. AWS offers on-demand cloud computing services to build your own web-based applications independent of university information technology bureaus.

EchinoDB contains amino acid sequence clusters of orthologous genes, termed orthoclusters. These orthoclusters were generated by RNA-Seq profiling of adult tissues from 42 echinoderm specimens representing 24 orders and 37 families from all five extant classes [19]. The RNA-Seq data was assembled using Trinity [44] and translated into peptides using Transdecoder [45]. The de novo transcriptome assembly consisted of 1,198,706 amino acid sequences across 42 species. The data was clustered using OrthoMCL, an algorithm for grouping orthologous protein sequences based on sequence similarity [46]. The resulting orthoclusters database consisted of groups of 749,397 orthologous and paralogous transcripts. These orthoclusters were annotated through sequence similarity using the genome of purple sea urchin *Strongylocentrotus purpuratus*, the best annotated echinoderm genome at the time of the origins of the project [47]. Complete RNA-Seq analysis pipeline (from RNA sampling and isolation to sequencing, de novo transcriptome assembly, translation, orthoclustering and annotation) was described in [19]. These annotated orthoclusters now provide the basis for keyword searches in EchinoDB.

### New data resources for ophiuroid and echinoid within the updated EchinoDB

We have added newly generated RNA-Seq data for *O. brevispinum* [16], a common brittle star found in shallow waters of the western Atlantic Ocean ranging from Canada to Venezuela. This resource can be found in EchinoDB under the name “OphiuroidDB”. We have also added the “EchinoidDB” resource that contains the high-quality genome assembly data of *L. variegatus* [20], a sea urchin found in shallow waters throughout the western Atlantic Ocean ranging from the United States to Venezuela. The rationale for creating these two new data resources is that there has been a growing use of these two species in recent molecular studies in developmental and regenerative biology [16, 20, 31, 48–52].

#### OphiuroidDB

We have provided the brittle star, *O. brevispinum* [22] transcriptome dataset, translated, and annotated using BLASTX [53] against the NCBI collection of predicted proteins of *S. purpuratus* [54] and protein models from UniProt’s Swiss-Prot [55] and NCBI’s RefSeq [56]. The application can be accessed via “Link to *O. brevispinum* transcriptome” in EchinoDB and is referred to as “OphiuroidDB”.

The transcriptome data of *O. brevispinum* were first used to characterize the downstream genes controlled by the Notch signaling pathway, which plays an important role in brittle star arm regeneration [16]. The raw sequencing reads of *O. brevispinum* transcriptome were submitted to the NCBI as a GEO dataset under the accession number GSE142391 [16, 57], and these sequences can now be also downloaded directly from OphiuroidDB. A total of 30,149 genes were identified, annotated, and included in the application.

#### EchinoidDB

EchinoidDB facilitates access to a recently published annotated high-quality chromosome-scale genome assembly of *L. variegatus* [20, 23]. The data *(Lvar_3.0)* includes 27,232 nucleotide and protein sequences, which were annotated using BLASTP [53] against UniProt Swiss-Prot [55], *S. purpuratus* [58] and non-*S. purpuratus* RefSeq invertebrate protein models [56]. These annotations can be downloaded from EchinoidDB.

## Utility and discussion

Echinoderms are a phylum of marine invertebrate deuterostomes and thus share a deep common ancestor with vertebrates [59–61]. However, unlike most vertebrates, many echinoderm species can regenerate all their tissue types after injury without developing cancers [17]. The capacity of adult echinoderms to fully regrow lost or damaged parts of their body is among the strongest in the animal kingdom [62]. The highly regenerative body parts include the central nervous system, digestive tube, connective tissue, epidermis, muscles, endoskeleton, and coelomic epithelial structures [2, 7, 10, 63]. However, the genomic and transcriptomic resources currently available today on echinoderms are limited to only a small fraction of species within the phylum. Most importantly, this data availability bias does not reflect the natural diversity in regenerative capacities among echinoderms. For example, the understudied sea cucumbers (class Holothuroidea) regenerate most of their organs [10, 14, 64–68], whereas sea urchins (class Echinoidea), which have been the main focus of the sequencing and annotation efforts so far, are weak in regeneration [49]. The web information systems that are currently available include Echinobase [69], HpBase [70], and SpBase [71]. These databases allow for the querying and exploration of the biological data mostly related to sea urchin and hence, they are not suitable for capturing much of the diversity of the phylum Echinodermata. To illustrate further, the Echinobase information system [69] (https://www.echinobase.org/entry) contains genomic information for eight echinoderm species, five of which are sea urchins – *Strongylocentrotus purpuratus* (purple sea urchin), *Strongylocentrotus fransciscanus* (red sea urchin), *Allocentrotus fragilis* (sea urchin), *L. variegatus* (green sea urchin), *Patiria miniata* (bat star), *Parastichopus parvimensis* (warty sea cucumber), *Ophiothrix spiculata* (spiny brittle star), and *Eucidaris tribuloides* (slate pencil urchin). Another commonly used resource, SpBase [71] (https://spbase.org/) is a system of databases that is mostly focused on sea urchin species and contains genomic information of *Strongylocentrotus purpuratus, Strongylocentrotus franciscanus, Allocentrotus fragilis, and L. variegatus*. Lastly, HpBase [70] contains genomic and transcriptomic information of a single sea urchin species, *Hemicentrotus pulcherrimus*. In contrast, EchinoDB contains biological data for 42 different echinoderm species representing all five echinoderm classes, in addition to transcriptomic and genomic data for *O. brevispinum* and *L. variegatus*. Thus, EchinoDB serves as a valuable information resource to represent the diversity within the phylum and facilitate studies of regenerative phenomenon that varies widely among echinoderms.

**Fig. 1:**
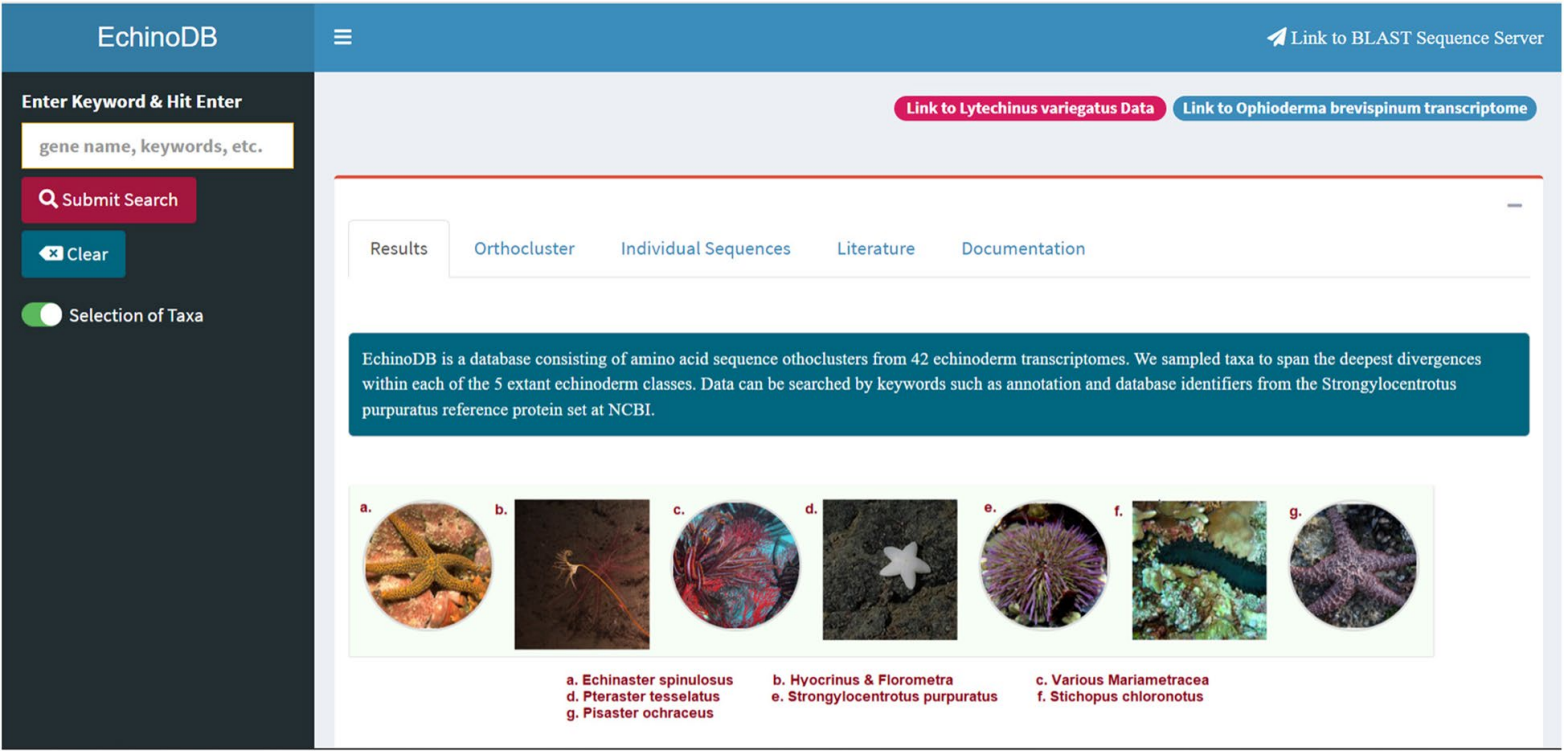
Screenshot of the EchinoDB landing page [18], available at https://echinodb.uncc.edu. Users can search against all echinoderm classes, orders, and families or un-toggle to retrieve information for a particular taxon

In the latest EchinoDB release, we added a text box that allows users to conduct searches using National Center for Biotechnology Information (NCBI) accession numbers and other keywords with or without the use of wildcard entries. Results include protein sequence(s), annotated description(s), known NCBI GenInfo Identifier (GI ids), and orthocluster(s). The annotations are assigned based on alignment of our sequences to the well-characterized protein sequence dataset of *Strongylocentrotus purpuratus* (i.e., sequences attributed to taxon 7668 in NCBI’s RefSeq, accessed in August 2012). These results can be further filtered by name or GenInfo Identifier (GI ids) in the search box in the top right corner. Additionally, users are able to expand or narrow their search based on taxonomic class, order, and family via toggle switches. Figure 1 depicts the design created in R Shiny for the EchinoDB application. Each row of the result table represents an orthocluster with the sequence similarity count or total hits. The number of hits is clickable, facilitating the viewing and downloading of related amino acid and nucleotide sequences in FASTA format.

### Use case examples

To demonstrate the utility of EchinoDB v2.0 and associated resources, we used them to retrieve genes associated with the Notch [72] and Wnt [73] signaling pathways. This is a biologically relevant example, as both these pathways are required for regeneration in echinoderms [16, 32]. Knowledge of the Notch and Wnt signaling pathways is important because they are highly conserved in the animal kingdom and regulate a variety of cellular processes, including proliferation, differentiation, fate specification, and cell death [74–77]. Recent studies indicate that inhibiting the Notch signaling pathway prevented the brittle stars from fully regenerating their arms [16, 31]. Furthermore, Wnt signaling pathway is a major regulator of development throughout the animal kingdom. This pathway plays an important role in early regenerative events, including cell division, cell dedifferentiation and apoptosis that contribute to intestinal regeneration in holothurians [62, 78–83]. For example, in sea cucumber *Apostichopus japonicus, Wnt6*, *Wnt7* (*Wnt* gene family), *Fzd7* (*Frizzled* gene family), and *Dvl* (*Dishevelled* gene family) are all significantly upregulated during the early stages of intestinal regeneration [28, 29]. Similarly, in *Holothuria glaberrima*, *Wnt9* is upregulated in early intestinal primordium [30]. Expression knockdown of *Wnt7* and *Dvl* significantly inhibits intestinal regrowth in sea cucumbers, implying that the canonical Wnt signaling is essential for visceral regeneration [29].

**Fig. 2:**
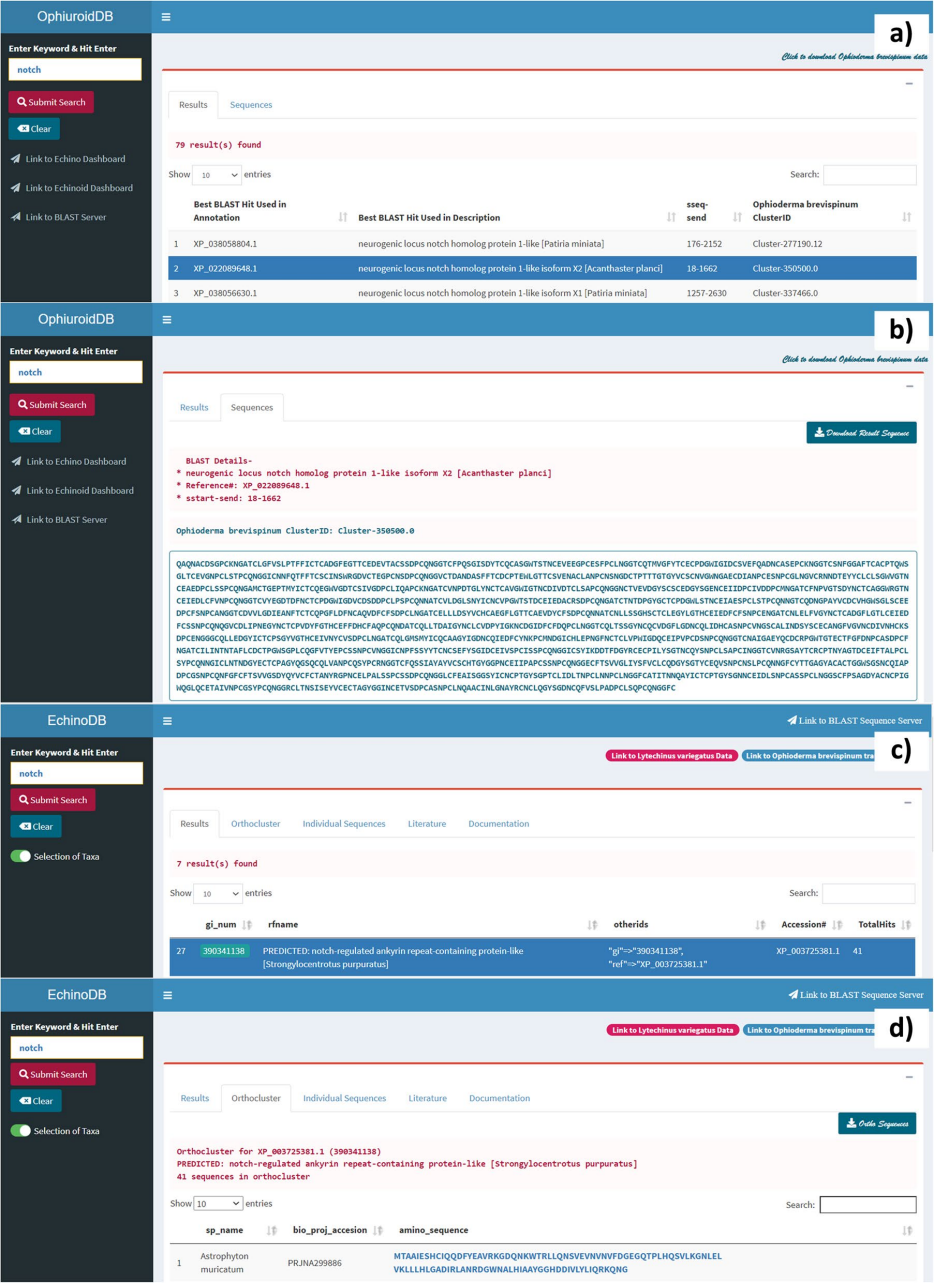
Usage example illustrating the search for Notch-related sequences in the brittle star *O. brevispinum* and other echinoderms. **a** Screenshot of the OphiuroidDB main page (https://echinodb.uncc.edu/BStarApp/) [22]. The image shows the results after searching for the keyword “Notch” against the database of the brittle star *O. brevispinum*. The interface allows the selection of any record on the results page to view the sequence. **b** Representative amino acid sequence from one selected Notch-related gene in OphiuroidDB. **c** Results after searching for the keyword “Notch” in EchinoDB (https://echinodb.uncc.edu) [18]. In this example, the search was conducted against the repository of clusters of orthologous genes discovered from echinoderm transcriptomes. A selected record will be highlighted, and amino acid sequences from the orthocluster repository will be displayed. **d** Amino acid sequence clusters of the selected orthologous record of the Notch-related gene from the EchinoDB repository

**Fig. 3:**
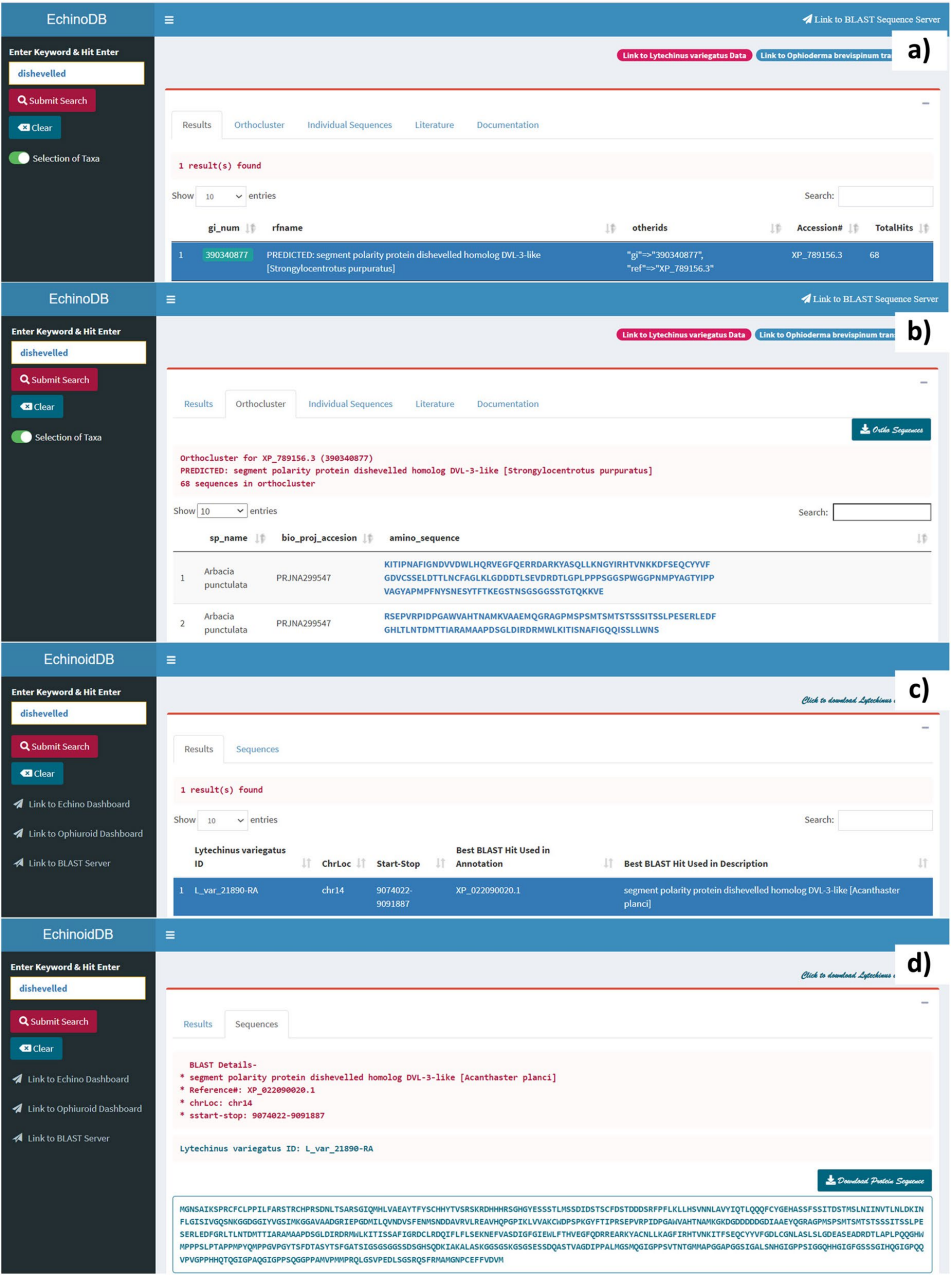
A use case illustrating the retrieval of the “*dishevelled*” gene from EchinoDB that contains orthocluster data from 42 different echinoderm species and EchinoidDB that contains biological data of the green sea urchin *L. variegatus. Dishevelled (Dvl)* gene functions as a principal component of the Wnt signaling pathway that governs several cellular processes, including cell proliferation, cell differentiation, and apoptosis or cell death. **a** Results after searching for the keyword “*dishevelled*” in EchinoDB (https://echinodb.uncc.edu) [18]. In this example, the search was conducted against the repository of clusters of orthologous genes discovered from echinoderm transcriptomes. A selected record will be highlighted, and amino acid sequences from the orthocluster repository will be displayed. **b** Displays amino acid sequence clusters of the selected orthologous record of the “*dishevelled*” gene group from the EchinoDB repository. **c** Screenshot of the EchinoidDB main page (https://echinodb.uncc.edu/SUrchinApp/) [23]. The image shows the results after searching for the keyword “*dishevelled*” against the database of the green sea urchin *L. variegatus*. The interface allows the selection of any record on the results page to view the sequence. **d** Example amino acid sequence from selected record in EchinoidDB

Figure 2 demonstrates the function of EchinoDB v2.0 and some of its outputs. The figure depicts the step-by-step process by locating individual sequences or clusters of Notch-related amino acid sequences in brittle stars and other echinoderms. For example, the user can search EchinoDB for Notch-related genes and obtain the corresponding sequences and metadata from our web resources. To do this, the user can search for the keyword “Notch” in our web resources to locate Notch-related sequences in brittle stars and other echinoderms. The results include NCBI’s accession numbers, other unique identifiers, descriptions of the gene or scaffold, start and end positions of regions of the gene or scaffold, and other details depending on the application used. For the keyword “notch”, a total of 432 amino sequences distributed throughout 7 orthoclusters were found in EchinoDB (amino acid sequence orthoclusters of 42 echinoderm transcriptomes), 54 in OphiuroidDB (transcriptomic data for the brittle star *O. brevispinum*), and 38 in EchinoidDB (genomic and peptide sequences for the green sea urchin *L. variegatus*). Similarly, Fig. 3 illustrates the step-by-step process of obtaining the corresponding sequences and metadata for *“dishevelled”* gene (*Dvl*) associated with the Wnt signaling pathway from our web resources. A total of 68 amino acid sequences found for “*dishevelled*” gene, grouped into a single orthocluster (XP_789156.3) in EchinoDB, four sequences were retrieved from OphiuroidDB and one from EchinoidDB. The search results corresponding to canonical Wnt and Notch signaling pathways are summarized in Tables 2 and 3.

**Table 2:** Key components of the Wnt signaling pathway retrieved from the database. For each gene, we list the gene name, the gene group it belongs to, and its role in the pathway. In addition, for each resource – EchinoDB, EchinoidDB, and OphiuroidDB – we show the number of sequences retrieved using keyword and BLAST search. Column search type represents K for keyword search and B for BLAST search. Number 0 indicates that no sequence was found in the database for that gene

Group	Gene Function	Name	Search Type	EchinoDB	EchinoidDB	OphiuroidDB	Cite
Regulator	Negative regulator. Part of the β-catenin destruction complex	APC	B	32	2	1	[73]
Regulator	Negative regulator. Part of the β-catenin destruction complex	Axin	K	54	1	2	[73]
Regulator	Phosphorylates β-catenin and the cytoplasmic tail of LRP. Part of the β-catenin destruction complex	CK1	B	225	4	2	[73]
Regulator	Negative regulator. Binds to LRP	Dickkopf	B	2	2	2	[73]
Regulator	Mediates the recruitment of Axin to the plasmalemma in the ON state of the pathway	Dishevelled	K	68	1	4	[73]
Receptor	Wnt receptors	Frizzled	B	500	8	7	[73, 84]
Regulator	Negative regulator. Transcriptional co-repressor. Binds to TCF in the OFF state of the pathway	Groucho	K	330	6	3	[73]
Regulator	Phosphorylates β-catenin and the cytoplasmic tail of LRP. Part of the β-catenin destruction complex	GSK3	K	56	1	0	[73]
Receptor	Dickkopf receptor. Mediates repression of the Wnt pathway	Kremen	B	413	45	49	[73, 85]
Regulator	Pathway enhancer. Receptor for R-spondin	Lgr5	B	500	1	4	[73]
Receptor	Wnt co-receptor	LRP	B	165	11	9	[73]
Ligand	Alternative ligand for the Wnt receptors	Norrin	B	3	0	0	[73]
Regulator	Negative regulator. Inactivates Wnt in the extracellular space through enzymatic action	Notum (Wingful)	K	43	1	0	[73, 86]
Modifier	Palmitoyl transferase, attaches palmitoleic acid to Wnt	Porcupine	K	23	1	1	[73]
Regulator	Negative regulator. Wnt target gene	Rnf43	B	189	7	9	[73]
Regulator	Pathway enhancer	R-spondin	K	12	1	1	[73]
Regulator	Negative regulator. Binds to LRP	Sclerostin	K	17	1	1	[73]
Regulator	Negative regulators. Sequester Wnts in the extracellular space	sFRPs	B	153	4	4	[84]
Transcription Factor	Transcriptional factors regulated by the Wnt pathway. Repress the target genes in the OFF state. Acitvate transctiption of the same genes in the ON state	TCF/Lef	K	48	2	1	[73]
Receptor	Norrin-specific co-receptor	Tspan12	B	146	5	4	[73]
Ligand	Paracrine/juxtacrine signaling molecules	Wnt	K	10	10	7	[73, 84]
Auxiliary protein	Specific intracellular transporter of Wnts	Wntless/Evi (Wls)	K	44	2	1	[73]
Regulator	Negative regulator. Wnt target gene	Znrf3	B	234	1	1	[73]
Regulator	Main modulator of the pathway	β-catenin	K	255	3	1	[73]
Regulator	Ubiquitinates the phosphorylate β-catenin thus targeting it for proteosomal destruction	β-TrCP	B	33	5	2	[73]

**Table 3:** Key components of the Notch signaling pathway retrieved from the database. Each line corresponds to the gene name, gene group, and its role in the pathway. In addition, we list the number of sequences retrieved from EchinoDB, EchinoidDB and OphiuroidDB using “keyword” and “BLAST” search. Column search type represents K for keyword search and B for BLAST search. Number 0 represents no sequence found in the corresponding database for that gene

Group	Gene Function	Name	Search Type	EchinoDB	EchinoidDB	OphiuroidDB	Cite
Motif	A disintegrin and metalloproteinase with thrombospondin motifs	ADAM 10/17	K	344	2	83	[72]
Receptor	Receptor proteolysis	Presenilin 1	K	77	2	3	[16, 72]
Transcription factor	HES-4-like	HES	K	46	3	3	[16]
Auxiliary protein	Mastermind-like protein. Co-activator of RBP-J	Mastermind	B	122	8	6	[72, 87]
Enzyme	E3 ubiquitin-protein ligase	Mindbomb	K	282	188	367	[16]
Protein coding	Notch Activation Complex Kinase. Co-activator of RBP-J	NACK	K	68	3	6	[16]
Transcription factor	CREB-binding protein. Co-activator of RBP-J	p300	K	103	2	2	[16]
Receptor	Neurogenic locus notch	Notch	K	14	35	50	[72, 88]
Receptor	Receptor proteolysis	Nicastrin	K	68	1	1	[72, 89]
Regulator	Negative regulator of the Notch pathway	Numb	K	68	0	1	[89]
Regulator	Context-dependent positive or negative regulator	Notchless	K	200	1	10	[90]
Regulator	Neuronal precursor cell-Expressed. Targets Notch and Deltex for degradation	Nedd4	K	94	2	2	[91]
Regulator	E3 ubiquitin-protein ligase/ DTX1. Context-dependent positive or negative regulator. Antagonizes Nedd4	Deltex	K	139	0	0	[74, 91]
Transcription factor	Mesoderm posterior bHLH transcription factor 2. Activates Fringe, induces degradation of Mastermind	Mesp2	B	6	2	1	[88]
Ligand	Ubiquitination of Jagged	Neuralized	K	159	6	26	[16]
Receptor	Ligand of the notch receptor	Delta/Serrate (Jagged)	K	68	2	2	[16]
Transcription factor	CBF1/ Recombination signal binding protein for immunoglobulin kappa J region. Transcription factor activated by Notch	RBP-J	K	1	1	0	[16, 72]
Regulator	Numb-associated kinase. Positive regulator of the Notch pathway	NAK	B	500	39	78	[16, 72]
Activator	Acyl-CoA-Binding Domain-Containing Protein 3. Activator of Numb	ACBD3	B	132	1	1	[16, 72]
Ligand	Ligand of Numb Protein 2. Negative regulator of Numb	LNX2	K	54	1	1	[72, 87]
Protein	Hairy/enhancer-of-split related with YRPW motif protein 1. Canonical target gene.	HEY1	K	24	3	1	[16]
Receptor	Paired basic amino acid cleaving enzyme. Receptor proteolysis	Furin	B	342	6	11	[16, 72]
Modifier	Protein O-glucosyltransferase. Post-translational maturation of Notch	Poglut	B	500	85	46	[16, 72]
Modifier	Protein O-fucosyltransferase 1. Post-translational maturation of Notch	POFUT1	K	191	5	1	[16, 72]
Modifier	beta-1,3-N-acetylglucosaminyltransferase radical fringe/ Lfng (lunatic) or Rfng (Radical). Post-translational maturation of Notch	Fringe	K	85	4	3	[16]
Repressor	SHARP/ spen family transcriptional repressor/ Mint/Sharp/SPEN, NCoR/SMRT, KyoT2. Co-repressor of RBP-J	MINT	B	95	1	3	[16, 72]
Repressor	Histone deacetylase 1. Co-repressor of RBP-J	HDAC1	K	220	1	0	[72]
Repressor	Nuclear receptor corepressor. Co-repressor of RBP-J	NCoR	B	77	2	0	[16, 72]
Repressor	Co-repressor interacting with RBP-J	CIR1	B	53	2	1	[16, 72]

In Table 2, we list the components of the canonical Wnt signaling pathway that were searched for in EchinoDB via a “keyword” search function. A number of orthoclusters, genomic, transcriptomic, and peptide sequences were found using this approach. A numerical value of 0 in the table indicates that no hits were returned when a particular gene name was used as a query for a “keyword” search in the database. However, the value 0 immediately raises a question: why are the sequences missing in our databases? For example, no matches are found in EchinoDB, when gene names “Kremen” and “Norrin” were used as keywords. Is it a limitation of the keyword search approach, a failure in annotation, or a true absence of homologs in EchinoDB? To answer this question, we conducted a test study, in which we performed a BLAST search (e-value cutoff 1e-06) [27, 26], instead of keyword search. For all the genes that were not retrieved by keyword search approach, we used reference sequences from the UniProt database [55] as a query in the BLAST search interface of EchinoDB. In all the cases, the genes that were not retrieved by keyword search were retrieved by BLAST search. Hence, in a case study of retrieving components of the Wnt signaling pathway, BLAST search and keyword search turned out to be two complementary strategies, with the former being more sensitive and the latter being faster but dependent on annotation quality of underlying data.

Another use case involved retrieving major components of the Notch pathway (i.e., the Notch receptor, the Delta and Serrate ligands, the transcriptional regulator RBPJ, two Notch target genes of the Hes family, and pathway modulators) [16, 72, 88–90]. As above, two complementary approaches were used to find all selected components of the Notch signaling pathway. First, we used a keyword search to retrieve sequences of all those genes of interest from EchinoDB and associated databases. Second, we used SequenceServer (BLAST) functionality in EchinoDB [33] to retrieve putative homologous sequences for the genes that were not retrieved by keyword search. The results of the keyword search and BLAST search are summarized in Table 3. Thus, BLAST search combined with keyword search proved useful in retrieving all major components of the Notch signaling pathway.

EchinoDB can also be used to expand our understanding of the clade-specific biology. For example, biomineralization contributes to the development of the stereome-type endoskeleton unique to echinoderms. Biomineralization is defined as the biologically controlled formation of mineral deposits resulting in structures that function as support, protection, or feeding anatomy [34]. Among echinoderms, biomineralization is best characterized in sea urchins [4]. Hence, we ask if we can use our database to obtain an insight on whether the biomineralization mechanisms described in echinoids are unique to that class or shared across the phylum. To this end, we leveraged the SequenceServer (BLAST search) functionality available within EchinoDB.

Among the proteins involved in biomineralization are spicule matrix proteins. In sea urchins, these secreted proteins are contained within the spicule and closely associated with the mineral component [4]. They have been shown to facilitate all aspects of endoskeleton formation, including nucleation of the crystal formation, as well as control of the orientation, shape and chemical purity of the resulting skeletal structure [35–37]. The spicule matrix protein family consists of nine members, including the most extensively studied SpSM50 and SpSM30B/C [4]. We used SequenceServer (BLAST) integrated in EchinoDB [33] with a cutoff e-value of 1e-06 to compare the amino acid sequences of the echinoid spicule matrix proteins against EchinoDB (42 species), OphiuroidDB (*O. brevispinum*) and EchinoidDB (*L. variegatus*). Table 4 lists a number of echinoid and non-echinoid species represented in EchinoDB that had a BLAST match to each of those nine reference echinoid spicule matrix proteins. All nine proteins had a putative ortholog in at least one non-echinoid class, which suggests that the skeletogenesis mechanisms discovered in sea urchins might be also shared by other members of the phylum.

**Table 4:** Spicule matrix proteins retrieved from the database. Each line corresponds to individual proteins, for which we list accession numbers of corresponding reference sequences from the NCBI, GenBank or UniProt databases. The numerical values in the table represent the number of species in each class of the phylum that had a BLAST match to the reference sequence

DataBase (Accession)	Protein	Description	Asteroidea	Ophiuroidea	Echinoidea	Holothuroidea	Crinoidea
NCBI (NP_999775.2)	SpSM50	50 kDa spicule matrix protein precursor [*Strongylocentrotus purpuratus*]	2	1	4	0	0
NCBI (NP_999776.1)	SpSM37	spicule matrix protein SM37 precursor [*Strongylocentrotus purpuratus*]	0	1	3	6	1
NCBI (NP_999803.1)	SpSM32	spicule matrix protein SM32 precursor *[Strongylocentrotus purpuratus*]	2	2	4	3	1
UniProt (P28163/SM30_STRPU)	SpSM30B/C	30 kDa spicule matrix protein precursor [*Strongylocentrotus purpuratus*]	4	1	4	1	0
NCBI (NP_999804.1)	SpSM29	spicule matrix protein SM29 precursor [*Strongylocentrotus purpuratus*]	2	0	4	0	0
GenBank (CAA42179.1)	LSM34	spicule matrix 34 kd protein [*Lytechinus pictus*]	2	2	4	1	0
UniProt (Q25116)	HSM30	30 kDa spicule matrix protein [*Hemicentrotus pulcherrimus*]	1	1	4	0	0
UniProt (Q26264)	HSM41	41 kDa spicule matrix protein [*Hemicentrotus pulcherrimus*]	2	2	4	0	0
UniProt (Q95W96)	PM27	Primary mesenchyme-specific protein [*Heliocidaris erythrogramma*]	1	3	3	3	0

Another echinoderm-specific phenomenon is the capacity of the connective tissue structures to rapidly change their tensile strength under the control of the central nervous system [5, 41, 42]. A subset of neurosecretory cells is thought to release proteins that can either stiffen or soften the extracellular collagenous matrix. Only one of such effector molecules, the TIMP-like protein tensilin has been characterized so far at the sequence level [92]. Tensilin, upon its release from the neurosecretory cells, stiffens the mutable collagenous tissue [39, 41, 42, 93]. Only three sequences are known thus far, all of them from members of the class Holothuroidea, including sea cucumbers *Cucumaria frondosa* [39], *Apostichopus japonicus* [94], and *Holothuria forskali* [40]. We therefore asked if tensilin, and thus tensilin-induced stiffening mechanisms, are unique to holothurians or are they represented in other classes of the phylum. To this end, we used the published protein and nucleotide sequences of tensilin as a query to perform BLASTP (for amino acid sequence) and BLASTX (for the nucleotide sequences) searches with an e-value threshold of 1e-06 [33]. This allows us to find potential homologs in species from all five echinoderm classes represented in our database, EchinoDB. The BLAST results are summarized in Table 5. They suggest that the tensilin protein, and thus the molecular mechanisms controlling the tensile strength of the mutable collagenous tissue, might be conserved across the phylum. This result is interesting groundwork for further study.

**Table 5:** Tensilin proteins. The first row corresponds to protein accession number from UniProt database whereas, second and third row depict nucleotide accession numbers from NCBI databases. The numerical values in the table represent the number of species in each class of the phylum that had a BLAST match to the reference sequence

DataBase (Accession)	Description	Asteroidea	Ophiuroidea	Echinoidea	Holothuroidea	Crinoidea
UniProt (Q962H0)	Tensilin [*Cucumaria frondosa*]	8	1	1	9	3
NCBI (KR002726.1)	*Apostichopus japonicus* tensilin mRNA, complete cds	5	1	2	9	0
NCBI (KY609179.1)	*Holothuria forskali* tensilin mRNA, complete cds	9	1	2	9	0

Finally, the database interface of EchinoDB allows the user to visualize any selected individual sequence or cluster of sequences or download them in FASTA format from the related repository. The downloaded sequences from EchinoDB v2.0 and associated resources can be used in downstream analyses (e.g. BRAKER [95, 96] or BLAST search for gene prediction and annotation in the draft genome of a newly sequenced echinoderm species). Alternatively, the sequences for any specific gene pathway from EchinoDB for example, Notch or Wnt, can be used in NCBI’s Conserved Domain Search (www.ncbi.nlm.nih.gov/Structure/cdd) to identity conserved protein domains in the sequences. The identified conserved domains can facilitate annotation of functionally unknown protein sequences. Hence, the above use cases illustrate how EchinoDB [18] in association with OphiuroidDB [22] and EchinoidDB [23] can be used to retrieve the gene sequences for cell signaling pathways essential in regeneration and facilitate better understanding of genomic underpinnings of phylum-specific biological phenomena. Further, EchinoDB can be used for sequence-similarity-based clustering analysis to get an insight about the conservation of various molecular components across echinoderms.

### Application features within updated EchinoDB

As many “omic” data for echinoderms are not yet well annotated, blast search is an important complement to keyword or accession search.

#### Using Sequenceserver to run BLAST

The updated EchinoDB contains an instance of Sequenceserver [27], a web-based BLAST server that supports sequence similarity searches against nucleotide and protein sequence databases. EchinoDB provides nucleotide and protein databases to be queried against user provided sequences to facilitate sequence similarity searches using default or user-selected parameters.

**Fig. 4:**
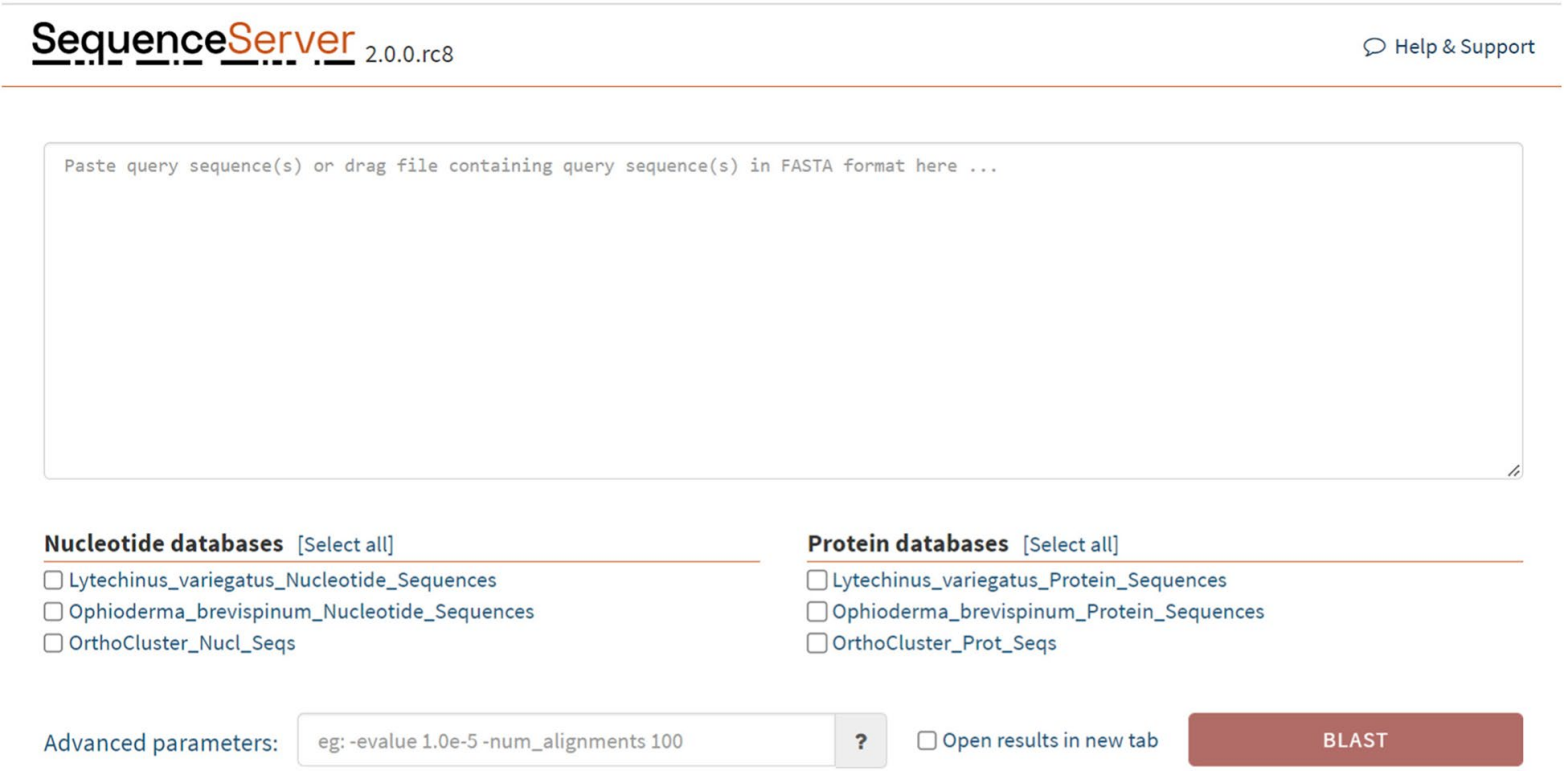
Screenshot of our Sequenceserver integrated in EchinoDB [33]. Users can perform BLAST searches against nucleotide and protein sequences of included datasets in the application via https://echinodb.uncc.edu/sequenceserver/

Integration with BLAST allows users of EchinoDB to search data resources with strings of the query sequence. Figure 4 illustrates Sequenceserver for BLAST functionality and can be accessed via “Link to BLAST Sequence Server” in the EchinoDB v2.0 application.

#### Literature

We provide a repository that contains links to many of the research papers associated with EchinoDB by their title. The literature repository is updated regularly.

#### Additional data

A link is added in the Literature section to allow users to download data associated with papers. For example, one dataset provides evidence that *Xyloplax* sp. is a velatid (an order within the class Asteroidea) asteroid rather than a new class [21]. The data included in EchinoDB includes tables and phylogenomic data from large amounts of transcriptome data used in this paper. The additional data repository is updated regularly.

#### Usage and documentation

EchinoDB, EchinoidDB, and OphiuroidDB user manuals (Additional files 2, 3 and 4: Files S1–3, respectively) are available in a tab named “Documentation” in the EchinoDB website. The user manuals are downloadable and provide instructions with screenshots to assist the user in navigating through the application.

## Conclusions

The updated EchinoDB provides, via a cloud-based server, additional tools and data from collaborations and our lab that can be of interest to a variety of scientific communities. One of our focal points in the future is to extend the genomic, transcriptomic, and orthocluster contents of EchinoDB.

## Supplementary Information

**Additional file 1: Table S1.** Raw reads from the various echinoderm species are available in NCBI’s SRA and is also available at Zenodo (doi: https://doi.org/10.5281/zenodo.6985492).

**Additional file 2: File S1.** EchinoDB user manual contains screenshots of the outputs to assist new users with the features and functionality of the application.

**Additional file 3: File S2.** EchinoidDB user manual contains instructions to help users with the resources and operations available in the application.

**Additional file 4: File S3.** OphiuroidDB user manual to describe operations and capabilities of the application.

**Additional file 5: File S4.** Source code (in R) for EchinoDB, EchinoidDB, and OphiuroidDB. We have also provided three R scripts one for each app.

## Data Availability

Assembled sequences and orthoclusters are available in EchinoDB (https://echinodb.uncc.edu) [18]. Raw reads from the various echinoderm species are available in NCBI’s SRA (see accession numbers in Additional file 1: Table S1). Additionally, the user manuals and code for EchinoDB v2.0, EchinoidDB, and OphiuroidDB are available as Additional file 2: File S1, Additional file 3: File S2, Additional file 4: File S3, and Additional file 5: File S4, respectively. Additional files are available in Zenodo (doi: 10.5281/zenodo.6985492).

## Abbreviations

BLAST: Basic Local Alignment Search Tool
AWS: Amazon Web Services
NCBI: National Center for Biotechnology Information
GEO: Gene Expression Omnibus
HTML: HyperText Markup Language
CSS: Cascading Style Sheets
RNA-Seq: RNA Sequencing
RefSeq: NCBI Reference Sequence Database
FASTA: FAST All
