# Supplementary material for: EchinoDB: an update to the web-based application for genomic and transcriptomic data on echinoderms
Source: BMC Genom Data. 2022 Oct 23;23:75. doi: 10.1186/s12863-022-01090-6 (PMC9590158; doi:10.1186/s12863-022-01090-6)
Supplement: Supplementary file 4 — Additional file 4: File S3. OphiuroidDB user manual to describe operations and capabilities of the application. [file 12863_2022_1090_MOESM4_ESM.pdf]

Ophiuroid Database (OphiuroidDB) User Manual

Author: Varnika Mittal, vmittal@uncc.edu
Date: Nov. 2, 2021

1. Access Ophiuroid DB Application

Click on the following link to access the application: https://echinodb.uncc.edu/BStarApp

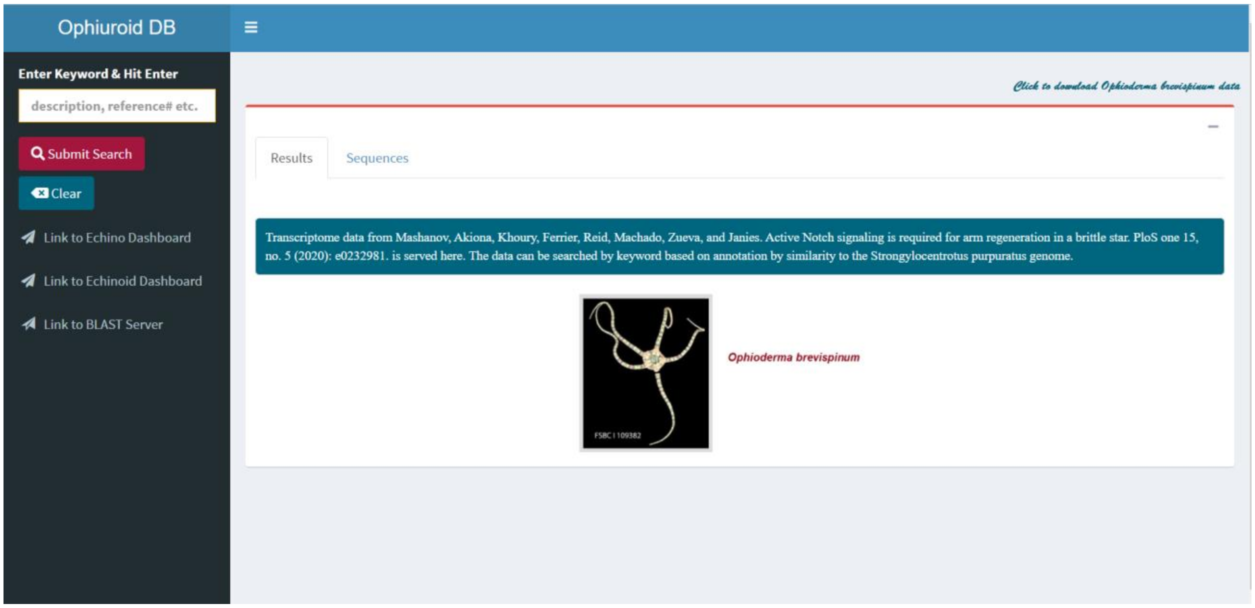

2. Perform Search against Transcriptome Data for *Ophioderma brevispinum* in Ophiuroid Database (Mashanov et al. 2020)

The user can search the transcriptome data of the brittle star *Ophioderma brevispinum* (Mashanov et al. 2020) in the Ophiuroid Database. Use any keyword or string such as "zinc", "chlor", "iron" or NCBI's Reference Number such as XP_0221120 for conducting search. Hit "Submit Search" button or "Enter" key in the upper left side of the web page.

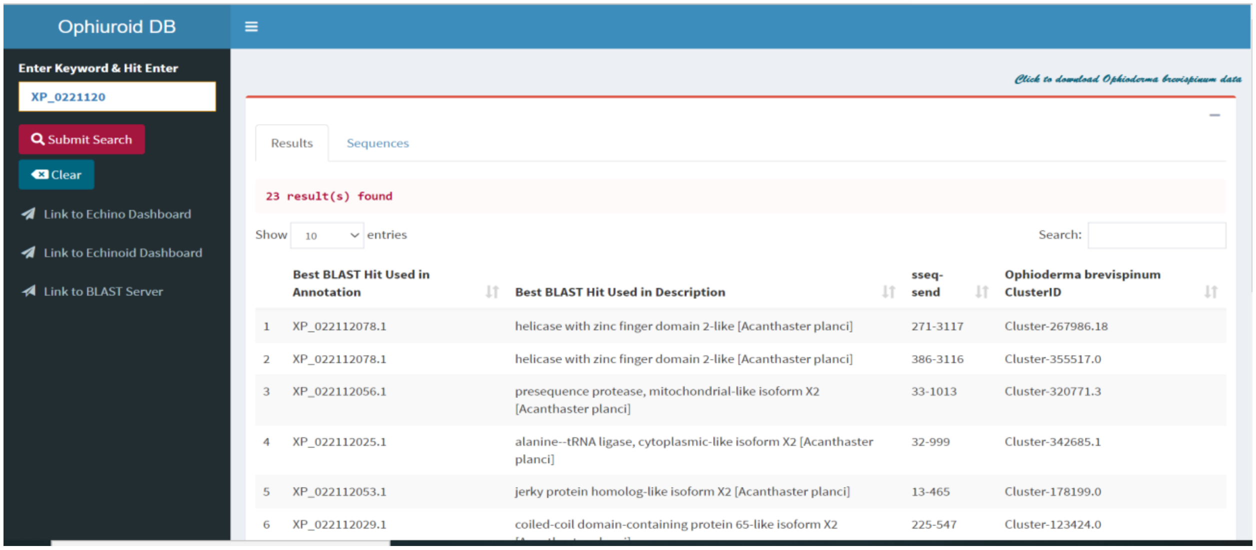

- ➢ With XP_0221120 keyword, 23 results are found.

- **Other Search Options**

  You can search by description or NCBI's Reference Number in the search box in top right corner.

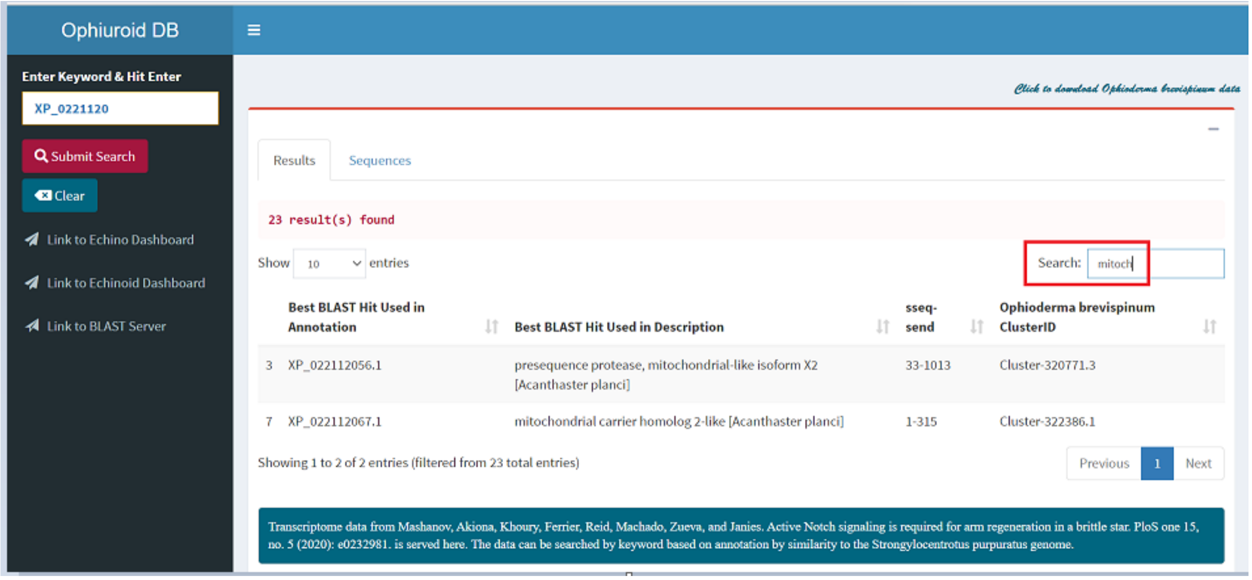

- **Downloading Search Results**
  A link “Click to download *Ophioderma brevispinum* data” is provided in the top right corner to download transcriptome sequences for *Ophioderma brevispinum* species.

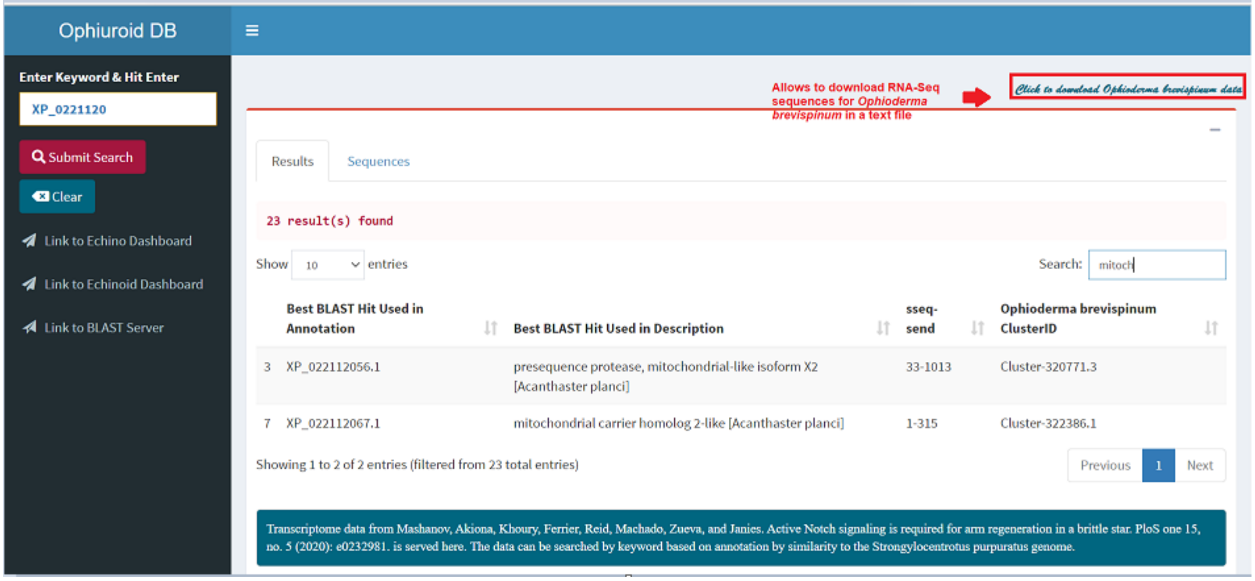

3. Visualizing Sequences

Select a whole row and the record will be highlighted with blue (you can only perform one selection at a time).

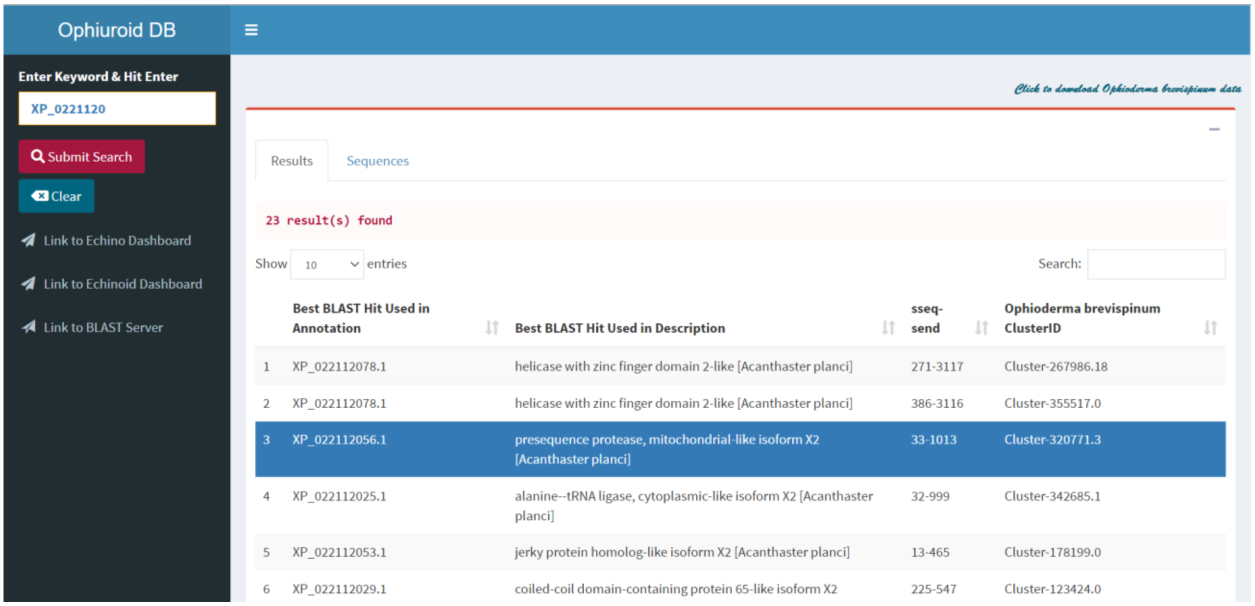

- **Sequences Tab**
  After the record is selected, it redirects you to "Sequences" tab and display protein sequences from Ophiuroids repository. Furthermore, it allows to download result sequence in fasta file format.

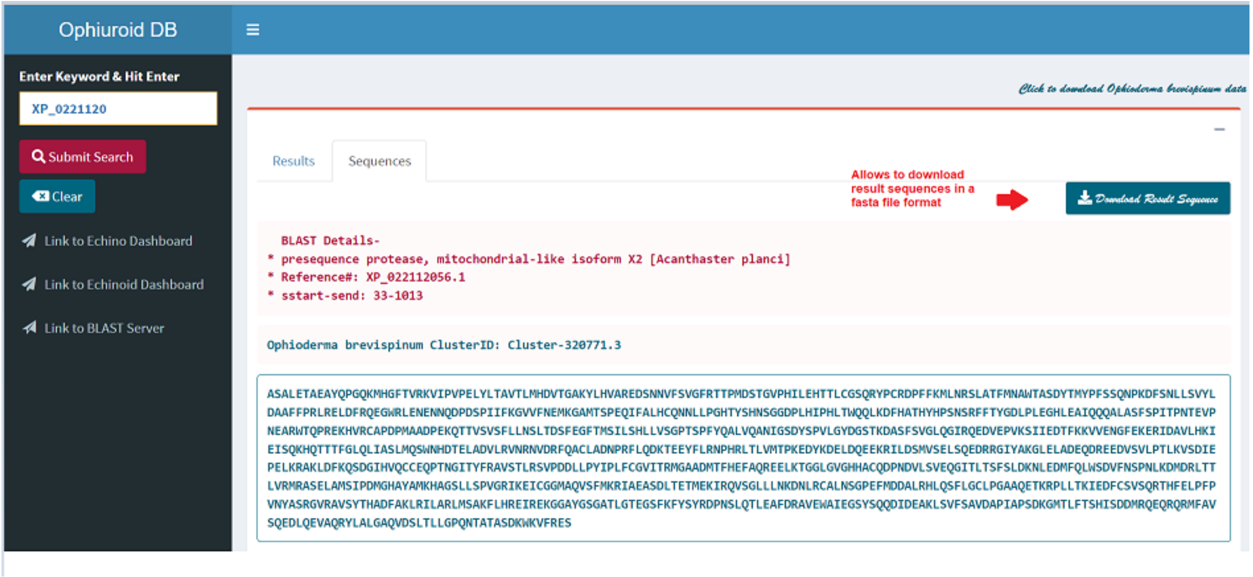

4. Sequenceserver for Basic Local Alignment Search Tool

Access Sequenceserver (Priyam *et al*. 2019) by clicking the "Link to BLAST Server" in the left pane or by type in the following URL: https://echinodb.uncc.edu/sequenceserver/

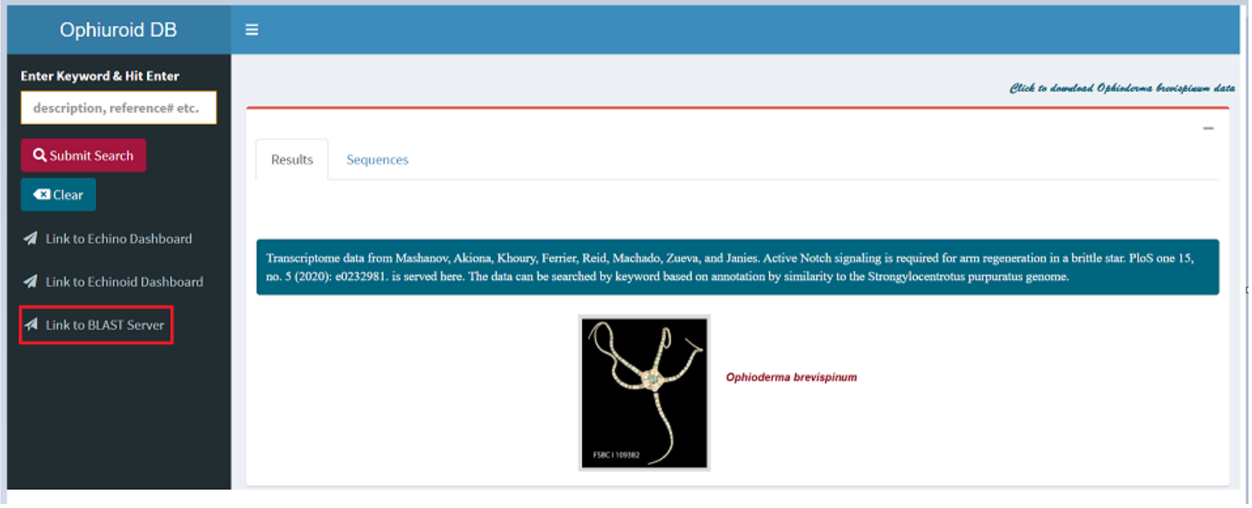

➢ Paste your query string [protein sequence(s)] in the text area to perform BLAST search.

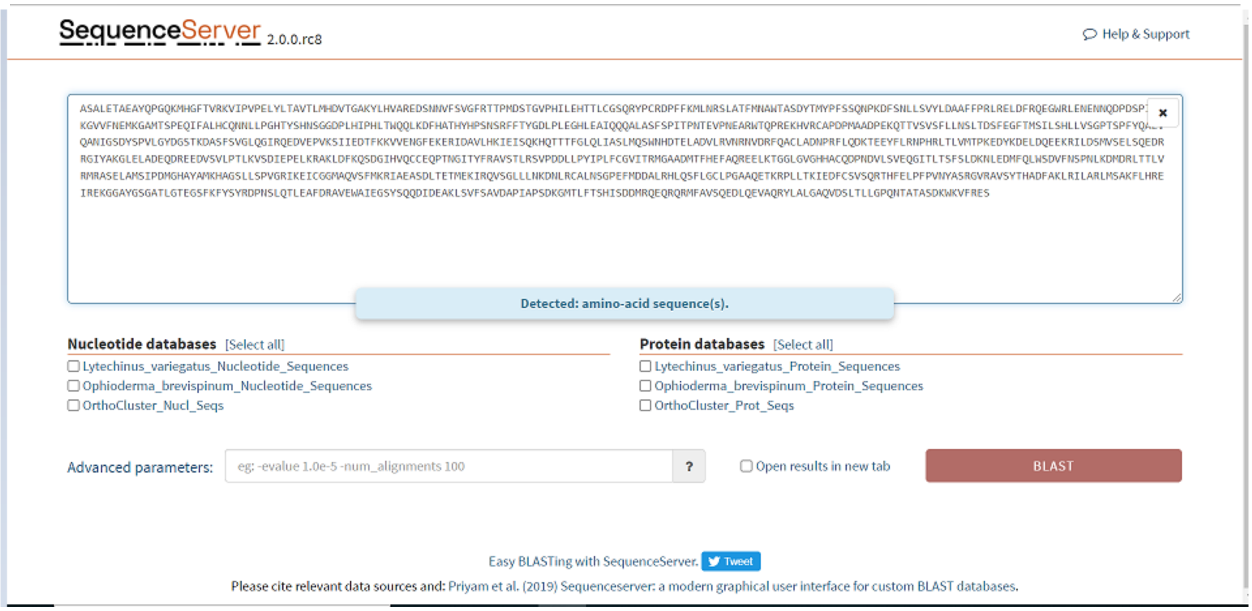

➢ Select database(s) to perform BLAST search against query sequence.

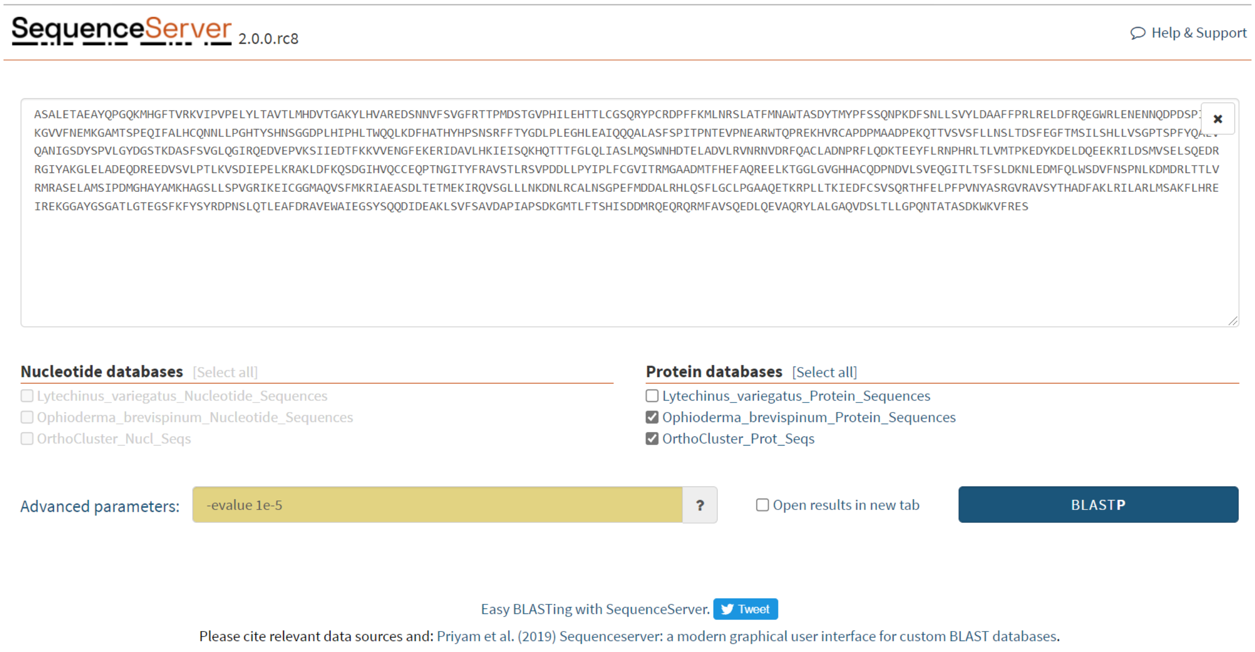

- ## Sequenceserver Results

SequenceServer 2.0.0.rc8 | Help & Support

BLASTP: 1 query, 2 databases
Edit search | New search

Download FASTA, XML, TSV
- FASTA of all hits
- FASTA of selected hit(s)
- Alignment of all hits
- Alignment of selected hit(s)
- Standard tabular report
- Full tabular report
- Full XML report

SequenceServer 2.0.0.rc8 using **BLAST 2.9.0+**, query submitted on 2021-09-30 01:49:53 UTC
Databases: Ophioderma_brevispinum_Protein_Sequences, OrthoCluster_Prot_Seqs (1228855 sequences, 299839407 characters)
Parameters: evalue 1e-05, matrix BLOSUM62, gap-open 11, gap-extend 1, filter F
Please cite: https://doi.org/10.1093/molbev/msz185

⊞ **Queries and their top hits: chord diagram**

Query= Query_1 length: 981

⊟ Graphical overview of hits | SVG | PNG

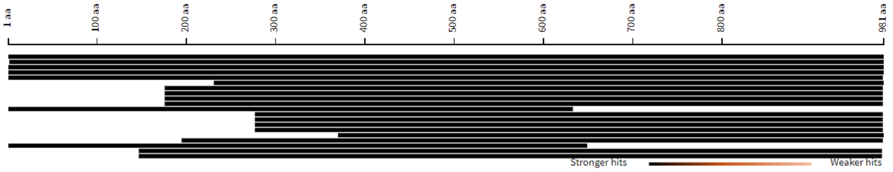

View More

⊞ Length distribution of hits
⊟ Summary table of hits

#	Similar sequences	Query coverage (%)	Total score	E value	Identity (%)
1.	Cluster-320771.3	100	5313	0	100
2.	20:16798 OS=Psilaster charcoti	99	4616	0	85
3.	19:33421 OS=Peribolaster BJ19 folliculatus	100	4445	0	82
4.	10:37131 OS=Pisaster ochraceus	100	4414	0	81
5.	13:53487 OS=Oligometra serripinna	99	3316	0	60
6.	22:9616 OS=Remaster gourdoni	77	3297	0	80
7.	7:93851 OS=Pteraster tesselatus	82	3244	0	72

- ## Download FASTA Sequence, Alignment from a high scoring pair from SequenceServer

⊟ **20:16798** OS=Psilaster charcoti hit 2, length: 1,003

☐ Select | Sequence | FASTA | Alignment
⊟ Graphical overview of aligning region(s) | SVG | PNG

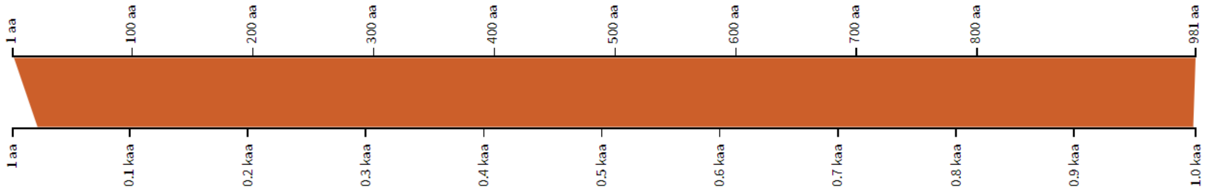


```
Score: 1782.69 (4616), E value: 0, Identity: 833/980 (85%), Positives: 907/980 (92.6%), Gaps: 0/980 (0%)

Query      2 SALETAEAYQPGQKMHGFTVRKVIPVPELYLTAVTLMHDVTGAKYLHVAREDSNNVFSVGFRTTPMDSTGVPHILEHTTLCGSQRYPCRDPFFKMLNR 99
             SAL+TAE Y+PGQK+HGFTV KVIPVPELYLTAV L HDVT AKYLHVAREDSNNVFSV FRTTPMDSTGVPHILEHTTLCGSQRYPCRDPFFKMLNR
Subject   22 SALKTAETYEPGQKLHGFTVEKVIPVPELYLTAVILTHDVTRAKYLHVAREDSNNVFSVSFRTTPMDSTGVPHILEHTTLCGSQRYPCRDPFFKMLNR 119

Query    100 SLATFMNAWTASDYTMYPFSSQNPKDFSNLLSVYLDAAFFPRLRELDFRQEGWRLENENNQDPDSPIIFKGVVFNEMKGAMTSPEQIFALHCQNNLLP 197
             SL+TFMNAWTASDYTMYPFSSQNPKDF NLLSVYLDAAFFPRLRELDFRQEGWRLENENNQDPDSPIIFKGVVFNEMKGAMTSPEQIFA HCQNNLLP
Subject  120 SLSTFMNAWTASDYTMYPFSSQNPKDFKNLLSVYLDAAFFPRLRELDFRQEGWRLENENNQDPDSPIIFKGVVFNEMKGAMTSPEQIFANHCQNNLLP 217

Query    198 GHTYSHNSGGDPLHIPHLTWQQLKDFHATHYHPSNSRFFTYGDLPLEGHLEAIQQQALASFSPITPNTEVPNEARWTQPREKHVRCAPDPMAADPEKQ 295
              HTYSHNSGGDPLHIP LTWQQLK+FHATHYHPSNSRFFTYGDLPLEGHLEAIQQQAL SFSPI+P+TEVPNE RWTQPREKHV CAPDPMAADPEKQ
Subject  218 SHTYSHNSGGDPLHIPRLTWQQLKEFHATHYHPSNSRFFTYGDLPLEGHLEAIQQQALESFSPISPDTEVPNETRWTQPREKHVHCAPDPMAADPEKQ 315

Query    296 TTVSVSFLLNSLTDSFEGFTMSILSHLLVSGPTSPFYQALVQANIGSDYSPVLGYDGSTKDASFSVGLQGIRQEDVEPVKSIIEDTFKKVVENGFEKE 393
             TTVSVSFLLNSL DSFEGFT+SILSHLLVSGPTSPFYQAL++ANIGSDYSPVLGYDGSTKDASFSVGLQGIR+EDVE VK II DTFKKVVE GFEKE
Subject  316 TTVSVSFLLNSLIDSFEGFTLSILSHLLVSGPTSPFYQALLEANIGSDYSPVLGYDGSTKDASFSVGLQGIREEDVEKVKGIIHDTFKKVVETGFEKE 413

Query    394 RIDAVLHKIEISQKHQTTTFGLQLIASLMQSWNHDTELADVLRVNRNVDRFQACLADNPRFLQDKTEEYFLRNPHRLTLVMTPKEDYKDELDQEEKRI 491
             RIDA+LHKIEISQKHQ+TTFGL L+ASLMQSWNHD +LA ++RVN+NV+RFQACLADNPRFLQDKTEEYF  N HRLTLVMTPKEDY + +DQEEKRI
Subject  414 RIDAILHKIEISQKHQSTTFGLNLVASLMQSWNHDADLASIMRVNKNVERFQACLADNPRFLQDKTEEYFTSNNHRLTLVMTPKEDYNETIDQEEKRI 511
```

5. Clear Search/Results in Ophiuroid DB

Go to the results tab and hit "Clear" button or "Delete" key to clear the search.

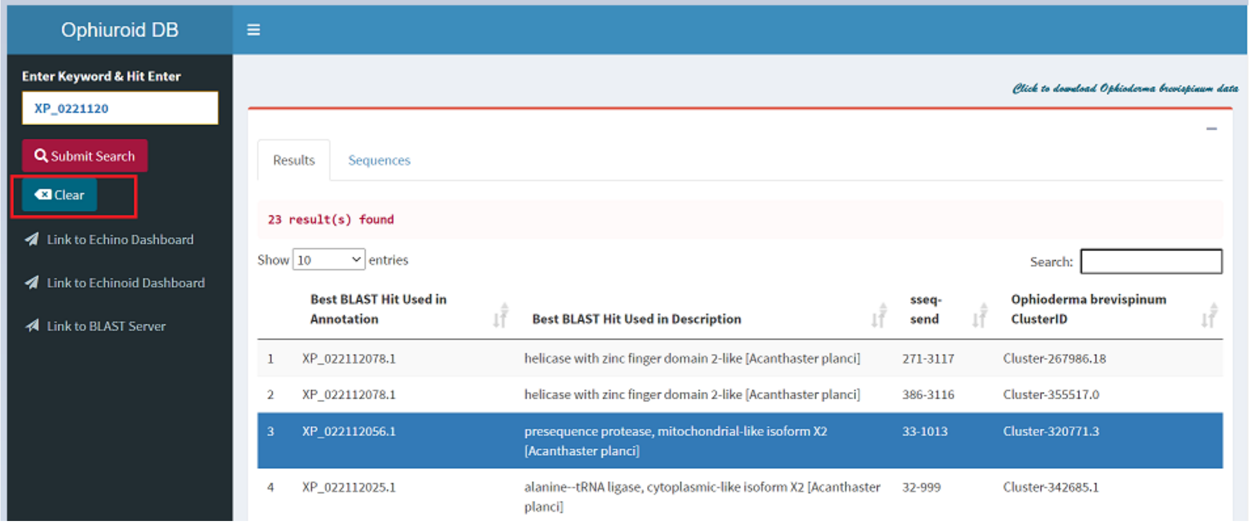

➢ And, search will be cleared after the button is clicked or delete key is pressed.

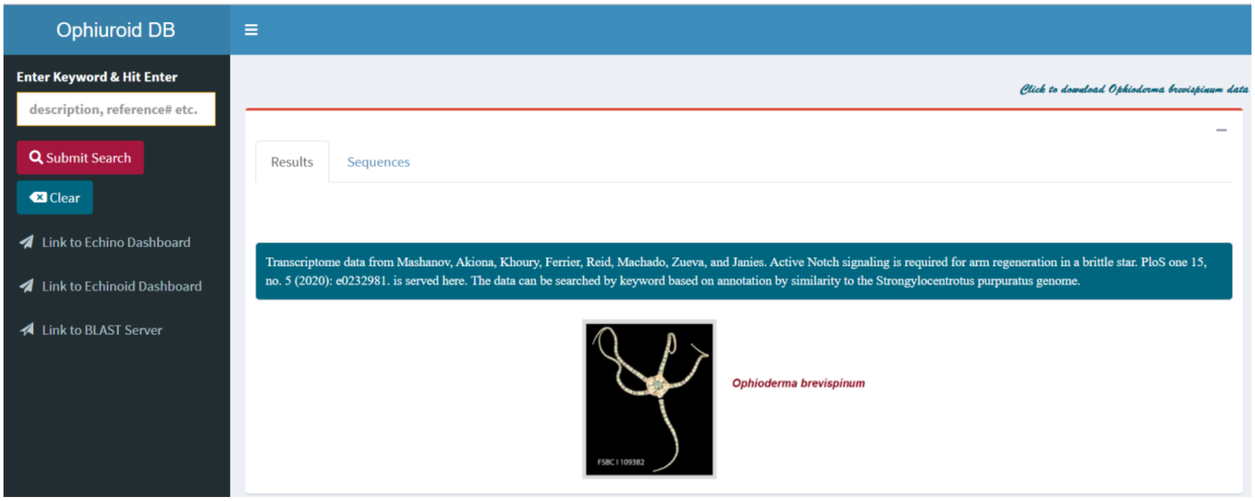

6. Additional Links

Links in the left pane are provided to redirect users to “EchinoDB” or “EchinoidDB” page.

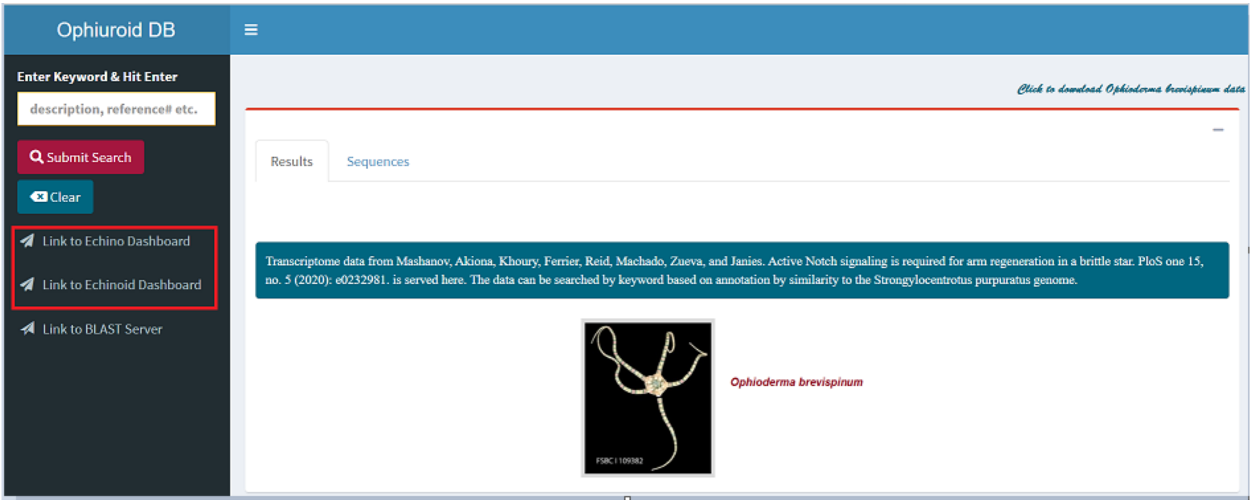

7. References

Mashanov V, Akiona J, Khoury M, Ferrier J, Reid R, Machado DJ, Zueva O, Janies D. Active Notch signaling is required for arm regeneration in a brittle star. *PLoS One*. 2020; **15(5)**:e0232981; doi: 10.1371/journal.pone.0232981.

Priyam A, Woodcroft BJ, Rai V, Moghul I, Munagala A, Ter F, Chowdhary H, Pieniak I, Maynard LJ, Gibbins MA, Moon H. Sequenceserver: a modern graphical user interface for custom BLAST databases. *Molecular Biology and Evolution*. 2019; **36(12)**: 2922-4. doi: doi.org/10.1093/molbev/msz185.
