# Supplementary material for: EchinoDB: an update to the web-based application for genomic and transcriptomic data on echinoderms
Source: BMC Genom Data. 2022 Oct 23;23:75. doi: 10.1186/s12863-022-01090-6 (PMC9590158; doi:10.1186/s12863-022-01090-6)
Supplement: Supplementary file 5 — Additional file 5: File S4. Source code (in R) for EchinoDB, EchinoidDB, and OphiuroidDB. We have also provided three R scripts one for each app. [file 12863_2022_1090_MOESM5_ESM.zip › FileS4_Source_Code.pdf]

EchinoDB (v2.0) code

```
library(shiny)
library(shinydashboard)
library(RPostgreSQL)
library(pool)
library(DBI)
library(DT)
library(formattable)
library(shinyWidgets)
library(seqinr)
library(shinyjs)

customGreen0 = "#ffffff"
customGreen = "#219f9c"
customBlue = "#337ab7"

setMethod("dbQuoteLiteral", c("Pool", "ANY"),
        function(conn, x, ...) {
          # As of 2020-05-07, this is necessary due to an incompatiblity
          # between packages `pool` (v 0.1.4.3) and `glue` (v >= 1.3.2).
          connection <- pool::poolCheckout(conn)
          on.exit(pool::poolReturn(connection))
          DBI::dbQuoteLiteral(connection, x, ...)
        }
)
# Loop through outp dataframe to extract reference seq id
# myfunc <- function(x){
#   y <- x[3]
#   s = gregexpr(pattern = 'XP_', y)
#   st <- s[[1]][1]
#   n<- nchar(y)
#   x[4] <- noquote(substring(y, st, n-1))
# }

concat <- function(x){
  aa <- x[1]
  bj <- x[2]
  sp <- x[3]
  y <- paste0(sp, ', aanum:', aa, ', bjnum:', bj)
  y
}

proper <- function(x) {
  x <- tolower(x)
  substr(x, 1, 1) <- toupper(substr(x, 1, 1))
  x
}
```

```
jscode <- '$(document).keyup(function(e) {
   if (e.key == "Enter") {
   $("#searchButton").click();
}});'

jsDeleteKey <- '$(document).keyup(function(e) {
   if (e.key == "Delete") {
   $("#clearButton").click();
}});'

#jsBackSpaceKey <- '$(document).keyup(function(e) {
#    if (e.key == "Backspace") {
#      $("#clearButton").click();
#}});'

jsRefresh <- 'shinyjs.refresh = function(){
  history.go(0);
}'

jsURL <- "
shinyjs.browseURL = function(url) {
  window.open(url,'_blank');
}
"
# Define UI for application
ui <- dashboardPage(
  dashboardHeader(title = "EchinoDB", tags$li(class = "dropdown", actionLink(inputId="ss", label="Link
to BLAST Sequence Server",
              class = "ss_class", icon = icon("send"), onclick
="window.open('https://echinodb.uncc.edu/sequenceserver/')"))),
  dashboardSidebar(
    tags$head(tags$style(HTML(".checkbox {margin: 0}
        .checkbox p {margin: 0;} .shiny-input-container {margin-bottom: 0;}" ))),
    tags$head(tags$style(HTML('#BriOptions,#ForciOptions,#NotoOptions,#PaxOptions,
        #SriOptions,#ValOptions,#VelOptions,#ComOptions,#HyoOptions,#ArbOptions,
        #CidOptions,#ClyOptions,#EchOptions,#ApoOptions,#AspOptions,#DenOptions,
        #DendOptions,#ElasOptions,#MolOptions,#EurOptions,#GnaOptions,#OphOptions,#OphiOptions
        {padding-top: 5px !important;border-color: #ffffff;border-style: dotted;color:#2E4053;background-
color:#FBFCFC;}'))),

    tags$head(tags$script(HTML(jscode))),
    tags$head(tags$script(HTML(jsDeleteKey))),
    #tags$head(tags$script(HTML(jsBackSpaceKey))),

    textInput(inputId = "searchText",placeholder = "gene name, keywords, etc.", label = "Enter Keyword &
Hit Enter"),
    actionButton(inputId = "searchButton", label="Submit Search",icon("search"),
```

```
            style="color: #fff; background-color: #a4163e; border-color:#a4163e;"),
   actionButton(inputId = "clearButton", label="Clear", icon("backspace"),
            style="color: #ffffff; background-color: #006680;border-color:#006680;"),

   prettySwitch(inputId = "allSwitch",label = "Selection of Taxa",fill = TRUE, status = "success", value =
TRUE),
   conditionalPanel(
    condition="input.allSwitch!=1",
    prettySwitch(inputId = "ASwitch",label = "Asteroidea",fill = TRUE, status = "info", value = TRUE),

    conditionalPanel(
     condition="input.ASwitch==1",
     checkboxGroupInput("Aoptions","Asteroidea:",
                choices=c("Brisingida",
"Forcipulatida","Notomyotida","Paxillosida","Spinulosida","Valvatida","Velatida"),
                selected=c("Brisingida",
"Forcipulatida","Notomyotida","Paxillosida","Spinulosida","Valvatida","Velatida")
     ),
     conditionalPanel("input.Aoptions.indexOf('Brisingida') > -1",
              checkboxGroupInput("BriOptions", "Brisingida",
                        choices=c("Brisingidae"),
                        selected= c("Brisingidae"))
     ),
     conditionalPanel("input.Aoptions.indexOf('Forcipulatida') > -1",
              checkboxGroupInput("ForciOptions", "Forcipulatida:",
                        choices = c("Asteriidae", "Labidiasteridae"),
                        selected = c("Asteriidae", "Labidiasteridae"))
     ),
     conditionalPanel(condition="input.Aoptions.indexOf('Notomyotida') > -1",
              checkboxGroupInput("NotoOptions", "Notomyotida:",
                        choices = c("Benthopectinidae"),
                        selected=c("Benthopectinidae"))
     ),
     conditionalPanel(condition="input.Aoptions.indexOf('Paxillosida') > -1",
              checkboxGroupInput("PaxOptions", "Paxillosida:",
                        choices = c("Astropectinidae", "Luidiidae"),
                        selected = c("Astropectinidae", "Luidiidae"))
     ),
     conditionalPanel("input.Aoptions.indexOf('Spinulosida') > -1",
              checkboxGroupInput("SriOptions", "Spinulosida:",
                        choices=c("Echinasteridae"),
                        selected= c("Echinasteridae"))
     ),
     conditionalPanel(condition="input.Aoptions.indexOf('Valvatida') > -1",
              checkboxGroupInput("ValOptions", "Valvatida:",
                        choices = c("Asteropseidae", "Poraniidae", "Solasteridae"),
                        selected = c("Asteropseidae", "Poraniidae", "Solasteridae"))
     ),
```

```
  conditionalPanel(condition="input.Aoptions.indexOf('Velatida') > -1",
           checkboxGroupInput("VelOptions", "Velatida:",
                   choices = c("Korethrasteridae", "Pterasteridae", "Xyloplacidae"),
                   selected = c("Korethrasteridae", "Pterasteridae", "Xyloplacidae"))
  )
 ),
 # Crinoidea Species and its order and family
 prettySwitch(inputId = "CSwitch",label = "Crinoidea",
       fill = TRUE, status = "info", value = TRUE),
 conditionalPanel(
  condition="input.CSwitch==1",
  checkboxGroupInput("Coptions","Crinoidea:",
            choices=c("Comatulida", "Hyocrinida"),
            selected=c("Comatulida", "Hyocrinida")
  ),
  conditionalPanel("input.Coptions.indexOf('Comatulida') > -1",
           checkboxGroupInput("ComOptions", "Comatulida:",
                   choices=c("Antedonidae", "Bourgueticrinidae", "Colobometridae",
"Comasteridae","Ptilometridae","Zenometridae"),
                   selected= c("Antedonidae", "Bourgueticrinidae", "Colobometridae",
"Comasteridae","Ptilometridae","Zenometridae"))
  ),
  conditionalPanel("input.Coptions.indexOf('Hyocrinida') > -1",
           checkboxGroupInput("HyoOptions", "Hyocrinida:",
                   choices=c("Hyocrinidae"),
                   selected= c("Hyocrinidae"))
  )
 ),
 # Echinoidea Species and its order and family
 prettySwitch(inputId = "ESwitch",label = "Echinoidea",
       fill = TRUE, status = "info", value = TRUE),
 conditionalPanel(condition="input.ESwitch==1",
          checkboxGroupInput("Eoptions","Echinoidea:",
                  choices=c("Arbacioida", "Cidaroida","Clypeasteroida", "Echinoida"),
                  selected=c("Arbacioida", "Cidaroida","Clypeasteroida", "Echinoida")),
          conditionalPanel("input.Eoptions.indexOf('Arbacioida') > -1",
                  checkboxGroupInput("ArbOptions", "Arbacioida:",
                          choices=c("Arbaciidae"),
                          selected= c("Arbaciidae"))
          ),
          conditionalPanel("input.Eoptions.indexOf('Cidaroida') > -1",
                  checkboxGroupInput("CidOptions", "Cidaroida:",
                          choices=c("Cidaridae"),
                          selected= c("Cidaridae"))
          ),
          conditionalPanel("input.Eoptions.indexOf('Clypeasteroida') > -1",
                  checkboxGroupInput("ClyOptions", "Clypeasteroida:",
                          choices=c("Dendrasteridae"),
```

```
                                    selected= c("Dendrasteridae"))
                  ),
                  conditionalPanel("input.Eoptions.indexOf('Echinoida') > -1",
                           checkboxGroupInput("EchOptions", "Echinoida:",
                                    choices=c("Strongylocentrotidae"),
                                    selected= c("Strongylocentrotidae"))
                  )
        ),
        # Holothuroidea Species and its order and family
        prettySwitch(inputId = "HSwitch",label = "Holothuroidea",
               fill = TRUE, status = "info", value = TRUE),
        conditionalPanel(
         condition="input.HSwitch==1",
         checkboxGroupInput("Hoptions","Holothuroidea:",
                  choices=c("Apodida", "Aspidochirotida","Dendrochirotacea", "Dendrochirotida",
"Elasipodida", "Molpadida"),
                  selected=c("Apodida", "Aspidochirotida", "Dendrochirotacea", "Dendrochirotida",
"Elasipodida", "Molpadida")
         ),
         conditionalPanel("input.Hoptions.indexOf('Apodida') > -1",
                  checkboxGroupInput("ApoOptions", "Apodida:",
                           choices=c("Synaptidae"),
                           selected= c("Synaptidae"))
         ),
         conditionalPanel("input.Hoptions.indexOf('Aspidochirotida') > -1",
                  checkboxGroupInput("AspOptions", "Aspidochirotida:",
                           choices=c("Stichopodidae", "Synallactidae"),
                           selected= c("Stichopodidae", "Synallactidae"))
         ),
         conditionalPanel("input.Hoptions.indexOf('Dendrochirotacea') > -1",
                  checkboxGroupInput("DenOptions", "Dendrochirotacea:",
                           choices=c("Cucumariidae", "Psolidae"),
                           selected= c("Cucumariidae", "Psolidae"))
         ),
         conditionalPanel("input.Hoptions.indexOf('Dendrochirotida') > -1",
                  checkboxGroupInput("DendOptions", "Dendrochirotida:",
                           choices=c("Psolidae"),
                           selected= c("Psolidae"))
         ),
         conditionalPanel("input.Hoptions.indexOf('Elasipodida') > -1",
                  checkboxGroupInput("ElasOptions", "Elasipodida:",
                           choices=c("Laetmogonidae"),
                           selected= c("Laetmogonidae"))
         ),
         conditionalPanel("input.Hoptions.indexOf('Molpadida') > -1",
                  checkboxGroupInput("MolOptions", "Molpadida:",
                           choices=c("Molpadidae"),
                           selected= c("Molpadidae"))
```

```
      )
    ),
    # Ophiuroidea Species and its order and family
    prettySwitch(inputId = "OSwitch",label = "Ophiuroidea", fill = TRUE, status = "info", value = TRUE),
    conditionalPanel(
      condition="input.OSwitch==1",
      checkboxGroupInput("Ooptions","Ophiuroidea:",
                 choices=c("Euryalida", "Gnathophiuridea", "Ophiocomidea", "Ophiurida"),
                 selected=c("Euryalida", "Gnathophiuridea","Ophiocomidea", "Ophiurida")
      ),
      conditionalPanel("input.Ooptions.indexOf('Euryalida') > -1",
                checkboxGroupInput("EurOptions", "Euryalida:",
                          choices=c("Gorgonocephalidae"),
                          selected= c("Gorgonocephalidae"))
      ),
      conditionalPanel("input.Ooptions.indexOf('Gnathophiuridea') > -1",
                checkboxGroupInput("GnaOptions", "Gnathophiuridea:",
                          choices=c("Ophiotrichidae"),
                          selected= c("Ophiotrichidae"))
      ),
      conditionalPanel("input.Ooptions.indexOf('Ophiocomidea') > -1",
                checkboxGroupInput("OphOptions", "Ophiocomidea:",
                          choices=c("Ophiocomidae"),
                          selected= c("Ophiocomidae"))
      ),
      conditionalPanel("input.Ooptions.indexOf('Ophiurida') > -1",
                checkboxGroupInput("OphiOptions", "Ophiurida:",
                          choices=c("Ophiodermatidae"),
                          selected= c("Ophiodermatidae"))
      )
    )
  )
 ),
 dashboardBody(
  shinyjs::useShinyjs(),
  shinyjs::extendShinyjs(text = jsRefresh, functions = c("refresh")),
  shinyjs::extendShinyjs(text = jsURL, functions = c("browseURL")),
  tags$head(tags$style(HTML('#searchText{color: #337ab7;border-color:orange; font-weight:bold;}'))),
  tags$style(type="text/css", "#text1{ height: 250px; color: #006680; background-color:#FFFFFF; border-
color:#006680; font-weight:bold; word-wrap: break-word; white-space: normal;}"),
  tags$style(type="text/css", "#text2, #text5{color: #b20838; background-color:#FFFAFA; font-
weight:bold; border-color:#FFFAFA;}"),
  tags$style(type="text/css", "#text{ background-color: #006680; color: #ffffff; word-wrap: break-word;
display: inline-block;
     overflow-wrap: break-word; word-break: normal;
     line-break: strict; white-space: normal; font-family: Times New Roman;}"),
  tags$style(type="text/css", "#text4{height: 80px; color: #006680; background-color:#FFFFFF; border-
color:#006680; font-weight:bold; word-wrap: break-word; white-space: normal;}"),
```

```
    tags$style(type="text/css", "#download, #download1, #download2, #downloadDoc{color: #ffffff;
background-color:#006680;float:right; font-family: Brush Script MT, monospace;}"),
    tags$style(type="text/css", "#text3{color: #b20838; background-color:#FFFAFA; font-weight:bold;
border-color:#FFFAFA;}"),
    tags$style(type="text/css", "#text6{color: #2F4F4F; font-weight:bold; font-family: Brush Script MT,
monospace;float:right;}"),
    tags$style(HTML(".ss_class {color: #FFFFFF; font-family: Copperplate; }")),
    tags$style(type="text/css", "#downloadFile{ font-weight:bold; font-family: Brush Script MT,
monospace;float:right;}"),
    tags$style(type = 'text/css',".badge{min-width: 200px;}"),
    tags$head(tags$style(HTML('.sidebar-menu>li>a{display: contents;}'))),

    sidebarMenu(
                menuItem(text= NULL,
          badgeColor = "light-blue", badgeLabel = "Link to Ophioderma brevispinum transcriptome",
          href = "https://echinodb.uncc.edu/BStarApp"),
                menuItem(text= NULL, badgeColor = "maroon", badgeLabel = "Link to Lytechinus variegatus
Data",
           href = "https://echinodb.uncc.edu/SUrchinApp")
    ),
    br(),
    br(),
    fluidRow(
      box(status = "danger", width=12, solidHeader = FALSE, collapsible = TRUE,
        tabsetPanel(id= "main",
                  tabPanel("Results", br(), verbatimTextOutput("text3"),
                         DT::dataTableOutput("table")),
                  tabPanel("Orthocluster", downloadButton('download2',"Ortho
Sequences"),br(),br(),verbatimTextOutput("text2"),
                         DT::dataTableOutput("table1")),
                  tabPanel("Individual Sequences", br(), verbatimTextOutput("text5"),
downloadButton('download',"Protein Sequence"),br(),br(), verbatimTextOutput("text4"),
                         downloadButton('download1', "DNA Sequence"), br(), br(),
verbatimTextOutput("text1")),
                  tabPanel("Literature", downloadLink('downloadFile',"Click for data from Linchangco et al.
2017"),br(), br(),
                         DT::dataTableOutput("papersTable")),
                  tabPanel("Documentation",
                         downloadButton('downloadDoc',"Download Document"),br(),br(),
                         textOutput("text6"),
                         DT::dataTableOutput("docsTable"))
        ),
        br(),
        verbatimTextOutput("text"),
        uiOutput("images")
      )
    )
```

```
  )
)

# Define server logic required to draw a histogram
server <- function(input, output, session) {
  pool <- dbPool(
    drv = dbDriver("PostgreSQL", max.con = 100),
    dbname = "XXXXXX",
    host = "XXXXXXXX",
    user = "XXXXXXX",
    password = "XXXXXXX",
    idleTimeout = 3600000
  )

  sqlP <- "SELECT title,link from echinopapers;"
  queryP <- sqlInterpolate(pool,sqlP)
  outLP <- dbGetQuery(pool, queryP)
  outLP1 <- as.data.frame(outLP[,1])
  colnames(outLP1) <- 'Title'

  sqlD <- "SELECT title,location, extension, contentTp from echinomanual;"
  queryD <- sqlInterpolate(pool,sqlD)
  outLD <- dbGetQuery(pool, queryD)
  outLD1 <- as.data.frame(outLD[,1])
  colnames(outLD1) <- 'Document Name'

  output$text <- renderText("EchinoDB is a database consisting of amino acid sequence othoclusters
from 42 echinoderm transcriptomes. We sampled taxa to span the deepest divergences within each of
the 5 extant echinoderm classes. Data can be searched by keywords such as annotation and database
identifiers from the Strongylocentrotus purpuratus reference protein set at NCBI.")
  output$images <- renderUI({
    tags$div(
                img(width= "80"),
                img(src='EchinoImages.PNG', width="800")
                  #img(src='Starfish.png', width="120", height="90"),
                  #img(width= "15"),
                  #img(src='Echino.png', width="120", height="90")
    )
  })
  observeEvent(input$allSwitch, {
    if(input$allSwitch == FALSE){
      updatePrettySwitch(session, inputId= "ASwitch", value = FALSE)
      updatePrettySwitch(session, inputId= "CSwitch", value = FALSE)
      updatePrettySwitch(session, inputId= "ESwitch", value = FALSE)
      updatePrettySwitch(session, inputId= "HSwitch", value = FALSE)
      updatePrettySwitch(session, inputId= "OSwitch", value = FALSE)
    } else if(input$allSwitch == TRUE){
      updatePrettySwitch(session, inputId= "ASwitch", value = TRUE)
```

```
      updatePrettySwitch(session, inputId= "CSwitch", value = TRUE)
      updatePrettySwitch(session, inputId= "ESwitch", value = TRUE)
      updatePrettySwitch(session, inputId= "HSwitch", value = TRUE)
      updatePrettySwitch(session, inputId= "OSwitch", value = TRUE)
    }}
  )

  observeEvent(input$searchButton, {
    req(input$searchText)
    sql <- "SELECT gi_num, rfname, otherids, refnum FROM refseqs_view WHERE ((rfname LIKE ?name OR
rfname LIKE ?name1 OR rfname LIKE ?name2) AND rfname NOT LIKE '%uncharacterized protein%') OR
(refnum LIKE ?name3);"
    query <- sqlInterpolate(pool,sql, name = noquote(paste0('%', tolower(input$searchText), '%')),
                         name1 = noquote(paste0('%', toupper(input$searchText), '%')),
                         name2 = noquote(paste0('%', proper(input$searchText), '%')),
                         name3 = noquote(paste0('%', input$searchText, '%')))

    outp <- dbGetQuery(pool, query)
    sql= NULL
    query = NULL

    if(length(outp) > 0) {
      if(input$allSwitch == TRUE || (input$allSwitch == FALSE & input$ASwitch == TRUE & input$CSwitch
== TRUE & input$ESwitch == TRUE & input$HSwitch == TRUE & input$OSwitch == TRUE)){
        v1 <- outp[,1]
        sql <- "SELECT COUNT(*),gi_num FROM orthoclusters WHERE gi_num IN ({gi*}) GROUP BY gi_num;"
        query <- glue::glue_sql(sql, gi = v1, .con=pool)
        outp1 <- dbGetQuery(pool, query)
        sql= NULL
        query = NULL

        outp <- as.data.frame(subset(outp, outp[,1] %in% outp1[,2]))

        # Sort outp and outp1 data by gi_num
        outp <- outp[order(outp$gi_num),]
        outp1 <- outp1[order(outp1[,2]),]

        # Create new column for total hits in outp dataframe
        outp$TotalHits <- outp1[,1]

        # Clear count data from ortholuster
        outp1 = NULL
        # names(outp)[names(outp) == "_searchtext"] <- "Refseq_Id"
        # outp[,4] <- apply(outp, 1, myfunc)
                 names(outp)[names(outp) == "refnum"] <- "Accession#"
        output$text3 <- renderText(paste0(nrow(outp), " result(s) found"))

        output$table <- DT::renderDataTable( {
```

```
    as.datatable(
      selection = "single",
      style = 'bootstrap',
      formattable(outp, list(
        paging = TRUE,cyl = col_format(),
        `gi_num` = color_tile(customGreen, customGreen)
      )))
  })


  # If row is selected, navigate to Orthocluster tab
  observeEvent(input$table_rows_selected, {
    shiny::withProgress(message='Please wait...', detail= 'Loading data', value = 0, {
      n <- 1
      for (i in 1:n) {
        shiny::incProgress(amount=1/n)
        selectedIndex <- input$table_rows_selected
        selectedRow <- outp[selectedIndex,]
        selectedGI <- selectedRow[,1]
        selectedHit <- paste0('Orthocluster for ', selectedRow[,4], ' (',
                              selectedGI, ')', '\n', selectedRow[,2], '\n',
                              selectedRow[,5], ' sequences in orthocluster')

        query <- sqlInterpolate(ANSI(),
                                "SELECT aanumber, bjnumber, concat(bjnumber, ':', aanumber) AS aa_bjnum,
gi_num from orthoclusters WHERE gi_num = ?gi_no;",
                                gi_no = selectedGI)
        outp2 <- dbGetQuery(pool, query)
        query <- NULL

        aabj = outp2[,3]

        sql1 <- "select aanumber, bjnumber, sp_name, bio_proj_accesion, amino_sequence from
TESTDB_VIEW where aa_bjnum IN ({aabj*});"
        query3 <- glue::glue_sql(sql1, aabj= aabj, .con=pool)
        outp3 <- dbGetQuery(pool, query3)
        sql1 <- NULL
        query3 <- NULL
        aabj = NULL

        # Generate Header for download orthocluster sequence
        seqs_fasta <- outp3[,5]
        giID <- paste0(', id:', selectedGI, ', orthocluster:', selectedRow[,4])
        namesList <- apply(outp3, 1, concat)
        names_fasta <- paste(namesList, rep(giID))
        namesList = NULL

        # Add space after 60 lines in a sequence
```

```
      outp3[,5] <- gsub('(.{60})', '\\1 ', outp3[,5])

      output$text2 <- renderText(selectedHit)
    } #End of Progress Bar For Loop
  })

  output$table1 <- renderDataTable({
    expr =  DT::datatable(outp3,
                selection="single",
                options = list(columnDefs = list(list(visible=FALSE, targets={1:2})))
    ) %>% formatStyle("amino_sequence", color = customBlue, fontWeight = 'bold')
  })

  output$download2 <- downloadHandler(
    filename = function() {
      paste("Ortho_Sequences-", Sys.Date(), ".fasta", sep="")
    },
    content = function(fname) {
      seqinr::write.fasta(as.list(seqs_fasta), names=names_fasta, file=fname)
    }
  )
  updateTabsetPanel(session, "main", selected = "Orthocluster")

  # If row is selected, navigate to tab3
  observeEvent(input$table1_rows_selected, {
    index <- input$table1_rows_selected
    aaNum = outp3[index,1]
    bjNum = outp3[index,2]
    amino_seq = gsub(" ", "", outp3[index,5])

    query2 <- sqlInterpolate(ANSI(),
                  "SELECT n.nuc_sequence FROM nucleotide2amino na INNER JOIN nucleotide n ON
(na.locustranscript = n.locustranscript AND na.bjnumber = n.bjnumber) WHERE na.bjnumber = ?bjNum
and na.aanumber = ?aaNum;",
                  bjNum = bjNum,
                  aaNum = aaNum
    )
    outp5 <- dbGetQuery(pool, query2)
    query2 = NULL

    output$text5 <- renderText(selectedHit)
    output$text4 <- renderText(paste0('Protein Sequence:', '\n',amino_seq))
    output$text1 <- renderText(outp5[,1])

    # Generate Header for download fasta file
    seq_header <- paste0(outp3[index,3], ',aanum:', aaNum, ' ,bjnum:', bjNum, giID)
    giID = NULL
```

```
          output$download <- downloadHandler(
            filename = function() {
              paste("Protein_Sequence-", Sys.Date(), ".fasta", sep="")
            },
            content = function(fname) {
              seqinr::write.fasta(as.list(amino_seq), names=seq_header, file=fname)
            }
          )
          output$download1 <- downloadHandler(
            filename = function() {
              paste("DNA_Sequence-", Sys.Date(), ".fasta", sep="")
            },
            content = function(fname) {
              seqinr::write.fasta(as.list(outp5[,1]), names=seq_header, file=fname)
            }
          )
          bjNum = NULL
          aaNum = NULL

          updateTabsetPanel(session, "main", selected = "Individual Sequences")
        })
      })
    } else {
      # Code to apply filters for one or more Echino Species
      if(input$ASwitch == TRUE & input$CSwitch == TRUE & input$ESwitch == TRUE & input$HSwitch ==
TRUE & input$OSwitch == FALSE){
        spList <- c("Asteroidea", "Crinoidea", "Echinoidea", "Holothuroidea")
        sp_order <- c(input$Aoptions, input$Coptions, input$Eoptions, input$Hoptions)
      } else if (input$ASwitch == TRUE & input$CSwitch == TRUE & input$ESwitch == TRUE &
input$HSwitch == FALSE & input$OSwitch == TRUE){
        spList <- c("Asteroidea", "Crinoidea", "Echinoidea", "Ophiuroidea")
        sp_order <- c(input$Aoptions, input$Coptions, input$Eoptions, input$Ooptions)
      } else if (input$ASwitch == TRUE & input$CSwitch == TRUE & input$ESwitch == FALSE &
input$HSwitch == TRUE & input$OSwitch == TRUE){
        spList <- c("Asteroidea", "Crinoidea", "Holothuroidea", "Ophiuroidea")
        sp_order <- c(input$Aoptions, input$Coptions, input$Hoptions, input$Ooptions)
      } else if (input$ASwitch == TRUE & input$CSwitch == FALSE & input$ESwitch == TRUE &
input$HSwitch == TRUE & input$OSwitch == TRUE){
        spList <- c("Asteroidea", "Echinoidea", "Holothuroidea", "Ophiuroidea")
        sp_order <- c(input$Aoptions, input$Eoptions, input$Hoptions, input$Ooptions)
      } else if (input$ASwitch == FALSE & input$CSwitch == TRUE & input$ESwitch == TRUE &
input$HSwitch == TRUE & input$OSwitch == TRUE){
        spList <- c( "Crinoidea", "Echinoidea", "Holothuroidea", "Ophiuroidea")
        sp_order <- c(input$Coptions, input$Eoptions, input$Hoptions, input$Ooptions)
      }

      else if (input$ASwitch == TRUE & input$CSwitch == TRUE & input$ESwitch == TRUE & input$HSwitch
== FALSE & input$OSwitch == FALSE){
```

```
        spList <- c("Asteroidea", "Crinoidea", "Echinoidea")
        sp_order <- c(input$Aoptions, input$Coptions, input$Eoptions)
      } else if (input$ASwitch == TRUE & input$CSwitch == TRUE & input$ESwitch == FALSE &
input$HSwitch == TRUE & input$OSwitch == FALSE){
        spList <- c("Asteroidea", "Crinoidea", "Holothuroidea")
        sp_order <- c(input$Aoptions, input$Coptions, input$Hoptions)
      } else if (input$ASwitch == TRUE & input$CSwitch == TRUE & input$ESwitch == FALSE &
input$HSwitch == FALSE & input$OSwitch == TRUE){
        spList <- c("Asteroidea", "Crinoidea", "Ophiuroidea")
        sp_order <- c(input$Aoptions, input$Coptions, input$Ooptions)
      } else if (input$ASwitch == TRUE & input$CSwitch == FALSE & input$ESwitch == TRUE &
input$HSwitch == TRUE & input$OSwitch == FALSE){
        spList <- c("Asteroidea", "Echinoidea", "Holothuroidea")
        sp_order <- c(input$Aoptions, input$Eoptions, input$Hoptions)
      } else if (input$ASwitch == TRUE & input$CSwitch == FALSE & input$ESwitch == TRUE &
input$HSwitch == FALSE & input$OSwitch == TRUE){
        spList <- c("Asteroidea", "Echinoidea", "Ophiuroidea")
        sp_order <- c(input$Aoptions, input$Eoptions, input$Ooptions)
      } else if (input$ASwitch == TRUE & input$CSwitch == FALSE & input$ESwitch == FALSE &
input$HSwitch == TRUE & input$OSwitch == TRUE){
        spList <- c("Asteroidea", "Holothuroidea", "Ophiuroidea")
        sp_order <- c(input$Aoptions, input$Hoptions, input$Ooptions)
      } else if (input$ASwitch == FALSE & input$CSwitch == TRUE & input$ESwitch == TRUE &
input$HSwitch == TRUE & input$OSwitch == FALSE){
        spList <- c("Crinoidea", "Echinoidea", "Holothuroidea")
        sp_order <- c(input$Coptions, input$Eoptions, input$Hoptions)
      } else if (input$ASwitch == FALSE & input$CSwitch == TRUE & input$ESwitch == TRUE &
input$HSwitch == FALSE & input$OSwitch == TRUE){
        spList <- c("Crinoidea", "Echinoidea", "Ophiuroidea")
        sp_order <- c(input$Coptions, input$Eoptions, input$Ooptions)
      } else if (input$ASwitch == FALSE & input$CSwitch == TRUE & input$ESwitch == FALSE &
input$HSwitch == TRUE & input$OSwitch == TRUE){
        spList <- c("Crinoidea", "Holothuroidea", "Ophiuroidea")
        sp_order <- c(input$Coptions, input$Hoptions, input$Ooptions)
      } else if (input$ASwitch == FALSE & input$CSwitch == FALSE & input$ESwitch == TRUE &
input$HSwitch == TRUE & input$OSwitch == TRUE){
        spList <- c("Echinoidea", "Holothuroidea", "Ophiuroidea")
        sp_order <- c(input$Eoptions, input$Hoptions, input$Ooptions)
      }

      else if (input$ASwitch == TRUE & input$CSwitch == TRUE & input$ESwitch == FALSE &
input$HSwitch == FALSE & input$OSwitch == FALSE){
        spList <- c("Asteroidea", "Crinoidea")
        sp_order <- c(input$Aoptions, input$Coptions)
      } else if (input$ASwitch == TRUE & input$CSwitch == FALSE & input$ESwitch == TRUE &
input$HSwitch == FALSE & input$OSwitch == FALSE){
        spList <- c("Asteroidea", "Echinoidea")
        sp_order <- c(input$Aoptions, input$Eoptions)
```

```
      } else if (input$ASwitch == TRUE & input$CSwitch == FALSE & input$ESwitch == FALSE &
input$HSwitch == TRUE & input$OSwitch == FALSE){
        spList <- c("Asteroidea", "Holothuroidea")
        sp_order <- c(input$Aoptions, input$Hoptions)
      } else if (input$ASwitch == TRUE & input$CSwitch == FALSE & input$ESwitch == FALSE &
input$HSwitch == FALSE & input$OSwitch == TRUE){
        spList <- c("Asteroidea", "Ophiuroidea")
        sp_order <- c(input$Aoptions, input$Ooptions)
      } else if (input$ASwitch == FALSE & input$CSwitch == TRUE & input$ESwitch == TRUE &
input$HSwitch == FALSE & input$OSwitch == FALSE){
        spList <- c("Crinoidea", "Echinoidea")
        sp_order <- c(input$Coptions, input$Eoptions)
      } else if (input$ASwitch == FALSE & input$CSwitch == TRUE & input$ESwitch == FALSE &
input$HSwitch == TRUE & input$OSwitch == FALSE){
        spList <- c("Crinoidea", "Holothuroidea")
        sp_order <- c(input$Coptions, input$Hoptions)
      } else if (input$ASwitch == FALSE & input$CSwitch == TRUE & input$ESwitch == FALSE &
input$HSwitch == FALSE & input$OSwitch == TRUE){
        spList <- c("Crinoidea", "Ophiuroidea")
        sp_order <- c(input$Coptions, input$Ooptions)
      } else if (input$ASwitch == FALSE & input$CSwitch == FALSE & input$ESwitch == TRUE &
input$HSwitch == TRUE & input$OSwitch == FALSE){
        spList <- c( "Echinoidea", "Holothuroidea")
        sp_order <- c(input$Eoptions, input$Hoptions)
      } else if (input$ASwitch == FALSE & input$CSwitch == FALSE & input$ESwitch == TRUE &
input$HSwitch == FALSE & input$OSwitch == TRUE){
        spList <- c( "Echinoidea", "Ophiuroidea")
        sp_order <- c(input$Eoptions, input$Ooptions)
      } else if (input$ASwitch == FALSE & input$CSwitch == FALSE & input$ESwitch == FALSE &
input$HSwitch == TRUE & input$OSwitch == TRUE){
        spList <- c( "Holothuroidea", "Ophiuroidea")
        sp_order <- c(input$Hoptions, input$Ooptions)
      }

      else if (input$ASwitch == TRUE & input$CSwitch == FALSE & input$ESwitch == FALSE &
input$HSwitch == FALSE & input$OSwitch == FALSE){
        spList <- c("Asteroidea")
        sp_order <- input$Aoptions
      } else if (input$ASwitch == FALSE & input$CSwitch == TRUE & input$ESwitch == FALSE &
input$HSwitch == FALSE & input$OSwitch == FALSE){
        spList <- c( "Crinoidea")
        sp_order <- input$Coptions
      } else if (input$ASwitch == FALSE & input$CSwitch == FALSE & input$ESwitch == TRUE &
input$HSwitch == FALSE & input$OSwitch == FALSE){
        spList <- c("Echinoidea")
        sp_order <- input$Eoptions
      } else if (input$ASwitch == FALSE & input$CSwitch == FALSE & input$ESwitch == FALSE &
input$HSwitch == TRUE & input$OSwitch == FALSE){
```

```
    spList <- c("Holothuroidea")
    sp_order <- input$Hoptions
  } else {
    spList <- c("Ophiuroidea")
    sp_order <- input$Ooptions
  }

  sp_familyA = sp_familyC = sp_familyE = sp_familyH = sp_familyO = NULL
  if(input$ASwitch == TRUE){
    tmp = tmp1 = tmp2 = tmp3 = tmp4 = tmp5 = tmp6 = NULL
    if('Brisingida' %in% input$Aoptions){
      tmp = input$BriOptions
    }
    if('Forcipulatida' %in% input$Aoptions){
      tmp1 = input$ForciOptions
    }
    if('Notomyotida' %in% input$Aoptions){
      tmp2 = input$NotoOptions
    }
    if('Paxillosida' %in% input$Aoptions){
      tmp3 = input$PaxOptions
    }
    if('Spinulosida' %in% input$Aoptions){
      tmp4 = input$SriOptions
    }
    if('Valvatida' %in% input$Aoptions){
      tmp5 = input$ValOptions
    }
    if('Velatida' %in% input$Aoptions){
      tmp6 = input$VelOptions
    }
    sp_familyA = c(tmp, tmp1, tmp2, tmp3, tmp4, tmp5, tmp6)
    tmp = tmp1 = tmp2 = tmp3 = tmp4 = tmp5 = tmp6 = NULL
  }

  if(input$CSwitch == TRUE){
    tmp = tmp1 = NULL
    if('Comatulida' %in% input$Coptions){
      tmp = input$ComOptions
    }
    if('Hyocrinida' %in% input$Coptions){
      tmp1 = input$HyoOptions
    }
    sp_familyC = c(tmp, tmp1)
    tmp = tmp1 = NULL
  }

  if(input$ESwitch == TRUE){
```

```
  tmp = tmp1 = tmp2 = tmp3 = NULL
  if('Arbacioida' %in% input$Eoptions){
    tmp = input$ArbOptions
  }
  if('Cidaroida' %in% input$Eoptions){
    tmp1 = input$CidOptions
  }
  if('Clypeasteroida' %in% input$Eoptions){
    tmp2 = input$ClyOptions
  }
  if('Echinoida' %in% input$Eoptions){
    tmp3 = input$EchOptions
  }
  sp_familyE = c(tmp, tmp1, tmp2, tmp3)
  tmp = tmp1 = tmp2 = tmp3 = NULL
}

if(input$HSwitch == TRUE){
  tmp = tmp1 = tmp2 = tmp3 = tmp4 = tmp5 = NULL
  if('Apodida' %in% input$Hoptions){
    tmp = input$ApoOptions
  }
  if('Aspidochirotida' %in% input$Hoptions){
    tmp1 = input$AspOptions
  }
  if('Dendrochirotacea' %in% input$Hoptions){
    tmp2 = input$DenOptions
  }
  if('Dendrochirotida' %in% input$Hoptions){
    tmp3 = input$DendOptions
  }
  if('Elasipodida' %in% input$Hoptions){
    tmp4 = input$ElasOptions
  }
  if('Molpadida' %in% input$Hoptions){
    tmp5 = input$MolOptions
  }
  sp_familyH = c(tmp, tmp1, tmp2, tmp3, tmp4, tmp5)
  tmp = tmp1 = tmp2 = tmp3 = tmp4 = tmp5 = NULL
}

if(input$OSwitch == TRUE){
  tmp = tmp1 = tmp2 = tmp3 = NULL
  if('Euryalida' %in% input$Ooptions){
    tmp = input$EurOptions
  }
  if('Gnathophiuridea' %in% input$Ooptions){
    tmp1 = input$GnaOptions
```

```
    }
    if('Ophiocomidea' %in% input$Ooptions){
      tmp2 = input$OphOptions
    }
    if('Ophiurida' %in% input$Ooptions){
      tmp3 = input$OphiOptions
    }
    sp_familyO = c(tmp, tmp1, tmp2, tmp3)
    tmp = tmp1 = tmp2 = tmp3 = NULL
  }

  sp_family = c(sp_familyA, sp_familyC, sp_familyE, sp_familyH, sp_familyO)
  # print(sp_family)

  sp_familyA = sp_familyC = sp_familyE = sp_familyH = sp_familyO = NULL
  # Convert array to a string
  p_class <- paste(unlist(spList), collapse=', ')

  if(length(sp_order) >= 1){
    p_order <- paste(unlist(sp_order), collapse=', ')
    if(length(sp_family) >= 1){
      p_family <- paste(unlist(sp_family), collapse=', ')
      # print(p_order)
      # print(p_family)
      qry1 <- pool %>% dbGetQuery(paste0("SELECT TESTDB_FUNC_SPECIES('{", p_class, "}', '{",
p_order, "}', '{", p_family, "}');"))
    } else {
      qry1 <- pool %>% dbGetQuery(paste0("SELECT TESTDB_FUNC_SPECIES('{", p_class, "}', '{",
p_order, "}', '{}');"))
    }
  } else {
    qry1 <- pool %>% dbGetQuery(paste0("SELECT TESTDB_FUNC_SPECIES('{", p_class, "}', '{}', '{}');"))
  }

  # Strip curly brackets off species function output
  qr <- gsub("\\{|\\}", "", qry1)
  # Convert string into array
  qr = strsplit(qr, ",")[[1]]

  # Query orthoclusters table based on bjnumber
  qry = "SELECT gi_num FROM orthoclusters WHERE bjnumber IN ({out*});"
  query_v <- glue::glue_sql(qry, out = qr, .con=pool)
  outp_view <- dbGetQuery(pool, query_v)
  # print(query_v)
  qry = NULL
  query_v = NULL

  outp = subset(outp, outp[,1] %in% outp_view[,1])
```

```
v1 = outp[,1]
outp_view = NULL

sql <- "SELECT COUNT(*),gi_num FROM orthoclusters WHERE gi_num IN ({gi*}) AND bjnumber IN
({bj*}) GROUP BY gi_num;"
query <- glue::glue_sql(sql, gi = v1, bj=qr , .con=pool)
outp1 <- dbGetQuery(pool, query)
sql = NULL
query = NULL

# Remove entries from outp based on gi_num
ifelse(length(outp[,1]) == 0, outp,
    {
     outp <- as.data.frame(subset(outp, outp[,1] %in% outp1[,2]))

     # Sort outp and outp1 data by gi_num
     outp <- outp[order(outp$gi_num),]
     outp1 <- outp1[order(outp1[,2]),]

     # Create new column for total hits in outp dataframe
     outp$TotalHits <- outp1[,1]

     # Clear count data from ortholuster
     outp1 = NULL
     # names(outp)[names(outp) == "_searchtext"] <- "Refseq_Id"
     # outp[,4] <- apply(outp, 1, myfunc)
     names(outp)[names(outp) == "refnum"] <- "Accession#"
    })
output$text3 <- renderText(paste0(nrow(outp), " result(s) found"))

# Table on first page to display data from orthoclusters based on ginum and bjnum
output$table <- DT::renderDataTable( {
  as.datatable(
    selection = "single",
    style = 'bootstrap',
    formattable(outp, list(
      paging = FALSE,cyl = col_format(),
      `gi_num` = color_tile(customGreen, customGreen)
    )))
})

# If row is selected, navigate to Orthoclusters Tab
observeEvent(input$table_rows_selected, {
  shiny::withProgress(message='Please wait...',
                      detail= 'Loading data',
                      value = 0, {
                        n <- 1
                        for (i in 1:n) {
```

```
                shiny::incProgress(amount=1/n)
                selectedIndex <- input$table_rows_selected
                selectedRow <- outp[selectedIndex,]
                selectedGI <- selectedRow[,1]
                selectedHit <- paste0('Orthocluster for ', selectedRow[,4], ' (',
                           selectedGI, ')', '\n', selectedRow[,2], '\n',
                           selectedRow[,5], ' sequences in orthocluster')

                sql = "SELECT aanumber, bjnumber, concat(bjnumber, ':', aanumber) AS aa_bjnum,
gi_num from orthoclusters WHERE gi_num IN ({gi*}) AND bjnumber IN ({bj*});"
                query <- glue::glue_sql(sql, gi = selectedGI, bj=qr , .con=pool)
                outp2 <- dbGetQuery(pool, query)
                sql = NULL
                query = NULL

                aabj = outp2[,3]
                sql1 <- "select aanumber, bjnumber, sp_name, bio_proj_accesion, amino_sequence
from TESTDB_VIEW where aa_bjnum IN ({aabj*});"
                query3 <- glue::glue_sql(sql1, aabj= aabj, .con=pool)
                outp3 <- dbGetQuery(pool, query3)
                sql1 = NULL
                query3 = NULL
                aabj = NULL

                # Generate Header for download orthocluster sequence
                seqs_fasta <- outp3[,5]
                giID <- paste0(', id:', selectedGI, ', orthocluster:', selectedRow[,4])
                namesList <- apply(outp3, 1, concat)
                names_fasta <- paste(namesList, rep(giID))
                namesList = NULL

                # Add space after 60 lines in a sequence
                outp3[,5] <- gsub('(.{60})', '\\1 ', outp3[,5])

                output$text2 <- renderText(selectedHit)
               } # End of Progress Bar For Loop
              })

    output$table1 <- renderDataTable({
     expr =  DT::datatable(outp3,
                selection="single",
                options = list(
                 columnDefs = list(list(visible=FALSE, targets={1:2})))
     ) %>% formatStyle("amino_sequence", color = customBlue, fontWeight = 'bold')
    })

    output$download2 <- downloadHandler(
     filename = function() {
```

```
      paste("Ortho_Sequences-", Sys.Date(), ".fasta", sep="")
    },
    content = function(fname) {
      seqinr::write.fasta(as.list(seqs_fasta), names=names_fasta, file=fname)
    }
  )
  updateTabsetPanel(session, "main", selected = "Orthocluster")

  # If row is selected, navigate to tab3
  observeEvent(input$table1_rows_selected, {
    index <- input$table1_rows_selected
    aaNum = outp3[index,1]
    bjNum = outp3[index,2]
    amino_seq = gsub(" ", "", outp3[index,5])

    query2 <- sqlInterpolate(ANSI(),
                "SELECT n.nuc_sequence FROM nucleotide2amino na INNER JOIN nucleotide n ON
(na.locustranscript = n.locustranscript AND na.bjnumber = n.bjnumber) WHERE na.bjnumber = ?bjNum
and na.aanumber = ?aaNum;",
                bjNum = bjNum,
                aaNum = aaNum)
    outp5 <- dbGetQuery(pool, query2)
    query2 = NULL

    output$text5 <- renderText(selectedHit)
    output$text4 <- renderText(paste0('Protein Sequence:', '\n',amino_seq))
    output$text1 <- renderText(outp5[,1])

    # Generate Header for download fasta file
    seq_header <- paste0(outp3[index,3], ',aanum:', aaNum, ' ,bjnum:', bjNum, giID)
    giID = NULL

    output$download <- downloadHandler(
      filename = function() {
        paste("Protein_Sequence-", Sys.Date(), ".fasta", sep="")
      },
      content = function(fname) {
        seqinr::write.fasta(as.list(amino_seq), names=seq_header, file=fname)
      }
    )
    output$download1 <- downloadHandler(
      filename = function() {
        paste("DNA_Sequence-", Sys.Date(), ".fasta", sep="")
      },
      content = function(fname) {
        seqinr::write.fasta(as.list(outp5[,1]), names=seq_header, file=fname)
      }
    )
```

```
      bjNum = NULL
      aaNum = NULL

      updateTabsetPanel(session, "main", selected = "Individual Sequences")
    })

   })
  }

 } else {
  outp <- NULL
  output$text3 <- renderText("No results to display...!!")
  updateTabItems(session, "main", "Results")
  output$table <- DT::renderDataTable(expr=NULL)
  output$table1 <- DT::renderDataTable(expr=NULL)
  output$table2 <- DT::renderDataTable(expr=NULL)
  output$text5 <- renderText("")
  output$text4 <- renderText("")
  output$text2 <- renderText("")
  output$text1 <- renderText("")
 }
})
observeEvent(input$clearButton, {
 shinyjs::js$refresh()
 updateTextInput(session, "searchText", value="")
 updateTabItems(session, "main", "Results")
 updatePrettySwitch(session, inputId= "allSwitch", value = TRUE)
 output$table <- DT::renderDataTable(expr=NULL)
 output$table1 <- DT::renderDataTable(expr=NULL)
 output$table2 <- DT::renderDataTable(expr=NULL)
 output$text5 <- renderText("")
 output$text4 <- renderText("")
 output$text3 <- renderText("")
 output$text2 <- renderText("")
 output$text1 <- renderText("")
})

output$papersTable <- DT::renderDataTable({
 as.datatable(
  selection = "single",
  style = 'bootstrap',
  formattable(outLP1, list(
   paging = TRUE,cyl = col_format())
  )
 )
})

# If row in papersTable is selected, Browse Literature Paper
```

```
observeEvent(input$papersTable_rows_selected, {
  ind = input$papersTable_rows_selected
  url = outLP[ind,2]
  # browseURL(url)
  js$browseURL(url)
})

#Link button to download Greg's Supplemental List Data
output$downloadFile <- downloadHandler(
  filename <- function() {
    paste("SupplementaryMaterials", "zip", sep=".")
  },
  content <- function(file) {
    file.copy("/home/ubuntu/EchinoManual/SupplementaryMaterials.zip", file)
  }
)

output$text6 <- renderText({ "Note: Please select document and press download button to save locally
in your downloads directory"})
output$docsTable <- DT::renderDataTable({
  shinyjs::disable("downloadDoc")
  as.datatable(
    selection = "single",
    style = 'bootstrap',
    options = list(dom = 't'),
    formattable(outLD1, list(
      paging=TRUE, cyl = col_format())
    )
  )
})
# If row in docsTable is selected, Download Documents
observeEvent(input$docsTable_rows_selected, {
  shinyjs::enable("downloadDoc")
  ind1 = input$docsTable_rows_selected
  fnameD = outLD[ind1,1]
  locD = outLD[ind1,2]
  extn = outLD[ind1, 3]
  contentTp = outLD[ind1, 4]
  print(locD)
  print(contentTp)
  output$downloadDoc <- downloadHandler(
    filename <- function() {
      paste(fnameD, extn, sep=".")
    },
    content <- function(file) {
      file.copy(locD, file)
    },
    contentType = contentTp
```

```
    )
  })


  col_format <- reactive({
    formatter("span",style = x ~ style(
      color = 'grey',font.weight = "bold"))
  })

  session$onSessionEnded(function() {
    pool::poolClose(pool)
  })

}

# Run the application
shinyApp(ui = ui, server = server)
```

EchinoidDB code

```
# load the required packages
library(shiny)
library(shinydashboard)
library(RPostgreSQL)
library(pool)
library(DBI)
library(DT)
library(formattable)
library(shinyWidgets)
library(shinyjs)

setMethod("dbQuoteLiteral", c("Pool", "ANY"),
          function(conn, x, ...) {
            # As of 2020-05-07, this is necessary due to an incompatiblity
            # between packages `pool` (v 0.1.4.3) and `glue` (v >= 1.3.2).
            connection <- pool::poolCheckout(conn)
            on.exit(pool::poolReturn(connection))
            DBI::dbQuoteLiteral(connection, x, ...)
          }
)

jscode <- '$(document).keyup(function(e) {
    if (e.key == "Enter") {
    $("#searchButton").click();
}});'

jsDeleteKey <- '$(document).keyup(function(e) {
```

```
    if (e.key == "Delete") {
    $("#clearButton").click();
}}});'

#Dashboard header carrying the title of the dashboard
header <- dashboardHeader(title = "EchinoidDB")

sidebar <- dashboardSidebar(
    #HTML CSS Scripts
    tags$head(tags$script(HTML(jscode))),
    tags$head(tags$script(HTML(jsDeleteKey))),

    tags$style(HTML('#searchText{color: #337ab7;border-color:orange; font-weight:bold;}')),
    tags$style(type="text/css", "#seqText {color: #006680;
        background-color:#FFFFFF; font-weight:bold; border-color:#006680;
        word-wrap: break-word; white-space: normal;}"
    ),

    tags$style(type="text/css", "#dnaSeq {height: 250px; color: #006680;
        background-color:#FFFFFF; font-weight:bold; border-color:#006680;
        word-wrap: break-word; white-space: normal;}"
    ),

    tags$style(type="text/css", "#descText{ background-color: #006680;
      color: #ffffff; word-wrap: break-word; display: inline-block;
      overflow-wrap: break-word; word-break: normal;
      line-break: strict; white-space: normal;
      font-family: Times New Roman;}"
    ),
    tags$style(type="text/css", "#infoText, #detailsText {color: #b20838;
        background-color:#FFFAFA; font-weight:bold; border-color:#FFFAFA;}"
    ),
    tags$style(type="text/css", "#detailsText1 {color: #006680;
        background-color:#FFFAFA; font-weight:bold; border-color:#FFFAFA;}"
    ),
    tags$style(type="text/css", "#downloadData{ font-weight:bold; color: #006680;
            font-family: Brush Script MT, monospace; float:right; padding: 9px;}"
    ),
    tags$style(type="text/css", "#dwnldResult, #dwnldDNA {color: #ffffff;
     background-color:#006680;float:right; font-family: Brush Script MT, monospace;}"),

    #Fields in Sidebar Pane
    textInput(inputId = "searchText",placeholder = "description, reference# etc.", label = "Enter Keyword
& Hit Enter"),
    actionButton(inputId = "searchButton", label="Submit Search",icon("search"),
              style="color: #fff; background-color: #a4163e; border-color:#a4163e;"),
    actionButton(inputId = "clearButton", label="Clear", icon("backspace"),
              style="color: #ffffff; background-color: #006680;border-color:#006680;"),
```

```
  sidebarMenu(
    menuItem("Link to Echino Dashboard", icon = icon("send"),
         href = "https://echinodb.uncc.edu"),
    menuItem("Link to Ophiuroid Dashboard", icon = icon("send"),
         href = "https://echinodb.uncc.edu/BStarApp/"),
    menuItem("Link to BLAST Server", icon = icon("send",lib='glyphicon'),
         href = "https://echinodb.uncc.edu/sequenceserver/")
  )
)

frow <- fluidRow(
  downloadLink('downloadData',"Click to download Lytechinus variegatus Data"),
  box(solidHeader = FALSE, status = "danger", width = 12, collapsible = TRUE,
    tabsetPanel(id= "main",
            tabPanel("Results", br(), verbatimTextOutput("infoText"),
                 DT::dataTableOutput("outpTable")),
            tabPanel("Sequences", br(), verbatimTextOutput("detailsText"),
verbatimTextOutput("detailsText1"),
              downloadButton('dwnldResult',"Download Protein Sequence"), br(), br(),
                        verbatimTextOutput("seqText"), downloadButton('dwnldDNA',"Download DNA
Sequence"),
              br(), br(), verbatimTextOutput("dnaSeq"))
    ),
       br(),
    verbatimTextOutput("descText"),
       uiOutput("images")
  )
)
# combine the two fluid rows to make the body
body <- dashboardBody(frow)

#completing the ui part with dashboardPage
ui <- dashboardPage(header, sidebar, body, skin='blue')

server <- function(input, output, session) {
  pool <- dbPool(
    drv = dbDriver("PostgreSQL", max.con = 100),
    dbname = "XXXXXX",
    host = "XXXXXXXX",
    user = "XXXXXXX",
    password = "XXXXXXX",
    idleTimeout = 3600000
  )
  output$descText <- renderText("A a high-quality, chromosomal-scale genome assembly
   data of the Sea Urchin Lytechinus variegatus
(https://academic.oup.com/gbe/article/12/7/1080/5841217) is served here. The data can be searched
   by keyword or downloaded from here."
  )
```

```
  output$images <- renderUI({
       tags$div(
         img(width= "80"),
     img(src='EchinoImages.PNG', width="800")
        )
  })

  observeEvent(input$searchButton, {
    req(input$searchText)
    sql <- "SELECT id, chromosome, startstop, ncbid, description FROM ly_variegatus WHERE (ncbid LIKE
?id) OR (description LIKE ?desc);"
    query <- sqlInterpolate(pool, sql, id = noquote(paste0('%', input$searchText, '%')),
                   desc = noquote(paste0('%', input$searchText, '%')))
    outp <- dbGetQuery(pool, query)
    sql= NULL
    query = NULL

    if(length(outp) > 0){
      output$infoText <- renderText(paste0(nrow(outp), " result(s) found"))
      output$outpTable <- DT::renderDataTable({
        colnames(outp) <- c("Lytechinus variegatus ID", "ChrLoc", "Start-Stop", "Best BLAST Hit Used in
Annotation",  "Best BLAST Hit Used in Description")
        as.datatable(
          selection = "single",
          style = 'bootstrap',
          formattable(outp, list(paging = TRUE))
        )
      })

      # If row is selected, navigate to Sequence Tab
      observeEvent(input$outpTable_rows_selected, {
        selectedIndex <- input$outpTable_rows_selected
        selectedRow <- outp[selectedIndex,]
        selectedID <- selectedRow[,1]      #Selected ID
        selectedChr <- selectedRow[,2]     #Selected Chromosome
        selectedSS <- selectedRow[,3]     #Selected Sequence Start-Stop
        selectedRef <- selectedRow[,4]     #Selected NCBI ID
        selectedDesc <- selectedRow[,5]     #Selected Description

        query <- sqlInterpolate(ANSI(),
              "SELECT a.protseq, a.dnaseq, b.description FROM lva_nuclprot_seq AS a INNER JOIN
l_var_fasta_header AS b ON a.id = b.id WHERE a.id = ?id;",                    id = selectedID)
        outp1 <- dbGetQuery(pool, query)
        query <- NULL

        protheader = paste0(outp1[3])
        nuclheader = gsub("protein", "transcript", outp1[3])
```

```
        #Header Text in Sequence Tab for BLAST Data
        output$detailsText <- renderText(paste0('  BLAST Details-\n', '* ', selectedDesc, '\n',
          '* Reference#: ', selectedRef, '\n',
          '* chrLoc: ', selectedChr, '\n', '* sstart-stop: ', selectedSS))

        output$detailsText1 <- renderText(paste0('Lytechinus variegatus ID: ', selectedID))
        output$seqText <- renderText(paste0(outp1[,1]))          #Resulting Protein Sequence

        output$dnaSeq <- renderText(paste0(outp1[,2]))  #Resulting Nucleotide Sequence

        output$dwnldResult <- downloadHandler(
           filename = function() {
              paste("LVA_Protein_Sequence_", Sys.Date(), ".fasta", sep="")
           },
           content = function(fname) {
              seqinr::write.fasta(outp1[,1], names=protheader, file=fname)
           }
        )

        output$dwnldDNA <- downloadHandler(
          filename = function() {
            paste("LVA_DNA_Sequence_", Sys.Date(), ".fasta", sep="")
          },
          content = function(fname) {
            seqinr::write.fasta(outp1[,2], names=nuclheader, file=fname)
          }
        )

        updateTabsetPanel(session, "main", selected = "Sequences")
      })
   }
   else {
      output$infoText <- renderText("No results to display...!!")
           output$outpTable <- DT::renderDataTable(expr=NULL)
   }
})

observeEvent(input$clearButton, {
   updateTextInput(session, "searchText", value="")
   updateTabItems(session, "main", "Results")
   output$seqText <- renderText("")
   output$infoText <- renderText("")
   output$detailsText <- renderText("")
   output$outpTable <- DT::renderDataTable(expr=NULL)
})

#Link button to download data
```

```
  output$downloadData <- downloadHandler(
    filename <- function() {
      paste("Lytechinus_variegatus_SuppData", "zip", sep=".")
    },
    content <- function(file) {
      file.copy("/home/ubuntu/EchinoManual/WrayData.zip", file)
    }
  )

  session$onSessionEnded(function() {
    pool::poolClose(pool)
  })
}
shinyApp(ui, server)
```

OphiuroidDB code

```
# load the required packages
library(shiny)
library(shinydashboard)
library(RPostgreSQL)
library(pool)
library(DBI)
library(DT)
library(formattable)
library(shinyWidgets)
library(shinyjs)

setMethod("dbQuoteLiteral", c("Pool", "ANY"),
 function(conn, x, ...) {
    # As of 2020-05-07, this is necessary due to an incompatiblity
    # between packages `pool` (v 0.1.4.3) and `glue` (v >= 1.3.2).
    connection <- pool::poolCheckout(conn)
    on.exit(pool::poolReturn(connection))
    DBI::dbQuoteLiteral(connection, x, ...)
 }
)

jscode <- '$(document).keyup(function(e) {
  if (e.key == "Enter") {
  $("#searchButton").click();
}});'

jsDeleteKey <- '$(document).keyup(function(e) {
  if (e.key == "Delete") {
  $("#clearButton").click();
}});'
```

```
#Dashboard header carrying the title of the dashboard
header <- dashboardHeader(title = "OphiuroidDB")
#Sidebar content of the dashboard
sidebar <- dashboardSidebar(
   #HTML CSS Scripts
   tags$head(tags$script(HTML(jscode))),
   tags$head(tags$script(HTML(jsDeleteKey))),
   tags$style(HTML('#searchText{color: #337ab7;border-color:orange; font-weight:bold;}')),

   tags$style(type="text/css", "#seqText { color: #006680;
      background-color:#FFFFFF; font-weight:bold; border-color:#006680;
      word-wrap: break-word; white-space: normal;}"
   ),
   tags$style(type="text/css", "#descText{background-color: #006680;
     color: #ffffff; word-wrap: break-word; display: inline-block;
     overflow-wrap: break-word; word-break: normal;
     line-break: strict; white-space: normal;
     font-family: Times New Roman;}"
   ),
   tags$style(type="text/css", "#infoText, #detailsText {color: #b20838;
      background-color:#FFFAFA; font-weight:bold; border-color:#FFFAFA;}"
   ),
   tags$style(type="text/css", "#detailsText1 {color: #006680;
      background-color:#FFFAFA; font-weight:bold; border-color:#FFFAFA;}"
   ),
   tags$style(type="text/css", "#downloadData{ font-weight:bold; color: #006680;
             font-family: Brush Script MT, monospace; float:right; padding: 9px;}"
   ),
   tags$style(type="text/css", "#dwnldResult {color: #ffffff;
    background-color:#006680;float:right; font-family: Brush Script MT, monospace;}"),

   #Fields in Sidebar Pane
   textInput(inputId = "searchText",placeholder = "description, reference# etc.", label = "Enter Keyword
& Hit Enter"),
   actionButton(inputId = "searchButton", label="Submit Search",icon("search"),
           style="color: #fff; background-color: #a4163e; border-color:#a4163e;"),
   actionButton(inputId = "clearButton", label="Clear", icon("backspace"),
           style="color: #ffffff; background-color: #006680;border-color:#006680;"),
   sidebarMenu(
      menuItem("Link to Echino Dashboard", icon = icon("send"),
           href = "https://echinodb.uncc.edu"),
             menuItem("Link to Echinoid Dashboard", icon = icon("send"),
           href = "https://echinodb.uncc.edu/SUrchinApp/"),
      menuItem("Link to BLAST Server", icon = icon("send",lib='glyphicon'),
           href = "https://echinodb.uncc.edu/sequenceserver/")
   )
)
```

```
frow <- fluidRow(
   downloadLink('downloadData',"Click to download Ophioderma brevispinum data"),
   box(solidHeader = FALSE, status = "danger", width = 12, collapsible = TRUE,
      tabsetPanel(id= "main",
         tabPanel("Results", br(), verbatimTextOutput("infoText"),
                DT::dataTableOutput("outpTable")),
         tabPanel("Sequences", downloadButton('dwnldResult',"Download Result Sequence"), br(), br(),
                      verbatimTextOutput("detailsText"),
            verbatimTextOutput("detailsText1"),
                      verbatimTextOutput("seqText"))
      ),
      br(),
            verbatimTextOutput("descText"),
      uiOutput("images")
   )
)
# combine the two fluid rows to make the body
body <- dashboardBody(frow)

#completing the ui part with dashboardPage
ui <- dashboardPage(header, sidebar, body, skin='blue')

server <- function(input, output, session) {
   pool <- dbPool(
      drv = dbDriver("PostgreSQL", max.con = 100),
      dbname = "XXXXXX",
            host = "XXXXXXXX",
            user = "XXXXXXX",
            password = "XXXXXXX",
      idleTimeout = 3600000
   )
   output$descText <- renderText("Transcriptome data from Mashanov, Akiona, Khoury, Ferrier, Reid,
Machado, Zueva, and Janies. Active Notch signaling is required for arm regeneration in a brittle
    star. PloS one 15, no. 5 (2020): e0232981. is served here.  The data can be searched by keyword
based on annotation by similarity to the Strongylocentrotus purpuratus genome."
   )
   output$images <- renderUI({
    tags$div(
      img(width= "320"),
            img(src='BStarImages.PNG', width="300")
    )
   })
   observeEvent(input$searchButton, {
      req(input$searchText)
      sql <- "SELECT accession, description, chrlocation, clusterid FROM ophiuroids WHERE (accession
LIKE ?rf) OR (description LIKE ?desc) ;"
      query <- sqlInterpolate(pool, sql, rf = noquote(paste0('%', input$searchText, '%')),
                  desc = noquote(paste0('%', input$searchText, '%')))
```

```
    outp <- dbGetQuery(pool, query)
    sql= NULL
    query = NULL

    if(length(outp) > 0){
      output$infoText <- renderText(paste0(nrow(outp), " result(s) found"))
      output$outpTable <- DT::renderDataTable({
        colnames(outp) <- c("Best BLAST Hit Used in Annotation", "Best BLAST Hit Used in Description",
"sseq-send", "Ophioderma brevispinum ClusterID")
        as.datatable(
          selection = "single",
          style = 'bootstrap',
          formattable(outp, list(paging = TRUE))
        )
      })

      # If row is selected, navigate to Sequence Tab
      observeEvent(input$outpTable_rows_selected, {
        selectedIndex <- input$outpTable_rows_selected
        selectedRow <- outp[selectedIndex,]
        selectedRef <- selectedRow[,1]      #Selected RefNum
                    selectedDesc <- selectedRow[,2]     #Selected Row Description
        selectedChr <- selectedRow[,3]      #Selected Chromosome Location
                    selectedCID <- selectedRow[,4]      #Selected Cluster ID

        query <- sqlInterpolate(ANSI(),
          "SELECT dnasequence FROM ophiuroids WHERE accession = ?rf AND chrlocation = ?chr AND
clusterid = ?cid;",
          rf = selectedRef, chr=selectedChr, cid=selectedCID)
        outp1 <- dbGetQuery(pool, query)
        query <- NULL

        # Store Sequence into a variable
        seq = outp1[1,1]

                    fastaheader = paste0(selectedCID, ',sp=Ophioderma brevispinum')

                    #Header Text in Sequence Tab for BLAST Data
                    output$detailsText <- renderText(paste0("  BLAST Details-", '\n', '* ', selectedDesc,
'\n',
                    '* Reference#: ', selectedRef, '\n', '* sstart-send: ', selectedChr))
        output$detailsText1 <- renderText(paste0('Ophioderma brevispinum ClusterID: ', selectedCID))

        output$seqText <- renderText(paste0(seq))   #Resulting Nucleotide or DNA Sequence

                    output$dwnldResult <- downloadHandler(
                    filename = function() {
```

```
                    paste("Ophioderma brevispinum Sequence _ ", Sys.Date(), ".fasta",
sep="")
                },
                content = function(fname) {
                    seqinr::write.fasta(seq, names=fastaheader, file=fname)
                }
        )

        updateTabsetPanel(session, "main", selected = "Sequences")
      })
    }
    else {
      output$infoText <- renderText("No results to display...!!")
          output$outpTable <- DT::renderDataTable(expr=NULL)
    }
  })

  observeEvent(input$clearButton, {
    updateTextInput(session, "searchText", value="")
    updateTabItems(session, "main", "Results")
    output$seqText <- renderText("")
    output$infoText <- renderText("")
        output$detailsText <- renderText("")
        output$detailsText1 <- renderText("")
    output$outpTable <- DT::renderDataTable(expr=NULL)
  })

  #Link button to download transcriptome data
  output$downloadData <- downloadHandler(
   filename <- function() {
    paste("Ophioderma Brevispinum Data", "txt", sep=".")
   },
   content <- function(file) {
    file.copy("/home/ubuntu/EchinoManual/Transcriptome_Data.txt", file)
   }
  )

  session$onSessionEnded(function() {
    pool::poolClose(pool)
  })
}
shinyApp(ui, server)
```
